# Time-varying associations of temperature and sleep as a marker of heat adaptation: A case study of Spanish outdoor workers over one summer of continuous monitoring

**DOI:** 10.1016/j.joclim.2026.100747

**Published:** 2026-10-17

**Authors:** Andrea Montanari, Constanza Vielma, Joan Ballester, Xavier Basagaña, Tarik Benmarhnia, Basile Chaix, Guillaume Chevance

**Affiliations:** a Sorbonne Université, INSERM, Institut Pierre Louis d’Epidémiologie et de Santé Publique, 75012, Paris, France; b ISGlobal, Barcelona, Spain; c Universitat Pompeu Fabra (UPF), Barcelona, Spain; d CIBER Epidemiología y Salud Pública (CIBERESP), Madrid, Spain; e Scripps Institution of Oceanography Department, University of California, San Diego, La Jolla, CA, United States; f Université de Rennes, Inserm, EHESP, Irset (Institut de Recherche en Santé, Environnement et Travail) - UMR_S, 1085, Rennes, France

**Keywords:** Sleep, Heat exposure, Heat stress, Outdoor workers, Accelerometer, Climate change

## Abstract

**Introduction:**

Rising global temperatures are increasingly recognized as a threat to sleep health. Outdoor workers, who often endure prolonged exposure to outdoor heat, may also be impacted by indoor nighttime temperatures. However, few studies have examined both exposures in this population, and, little is known about potential adaptation to heat in relation to sleep outcomes.

**Methods:**

We conducted an intensive longitudinal study with 13 Spanish outdoor workers over a 3-month summer period, during which sleep outcomes (i.e., total sleep time [TST], sleep efficiency [SE], and subjective sleep quality) were measured daily via an activity monitor and ecological momentary assessments. Temperatures were monitored both indoors and outdoors, using bedroom sensors and weather station data. Linear mixed-effects models were used to quantify the daily associations between heat and sleep.

**Results:**

Across the whole summer, each 1 °C increase in outdoor and indoor temperature led respectively to decreases of 2.13 (95% CI: −3.84, −0.42) and 3.18 (95% CI: −5.67, −0.70) minutes in total sleep time (TST); 0.09 (95% CI: −0.17, −0.01) and 0.18 (95% CI: −0.30, −0.07) percentage points in sleep efficiency (SE); and 0.09 (95% CI: −0.14, −0.04) and 0.17 (95% CI: −0.24, −0.09) points in subjective sleep quality. Time-varying analyses revealed that the adverse effects of heat on subjective sleep quality were attenuated in August compared with June and July for similar temperatures.

**Discussion:**

We found that both indoor and outdoor temperatures were associated with poorer sleep outcomes during the whole summer, along with preliminary evidence of reduced heat-related effects on subjective sleep quality later in the summer.

## Highlights

• Continuous monitoring of outdoor workers throughout the summer revealed that heat consistently impaired both objective and subjective sleep measures under real-world conditions.
• Indoor nighttime temperature showed stronger associations with sleep outcomes than outdoor temperature, underscoring the relevance of indoor exposure in heat–sleep research.
• Identifying time-varying temperature–sleep associations offer a novel framework for studying short-term human adaptation to heat.
• The adverse effects of temperature on subjective sleep quality weakened by late summer, suggesting potential adaptation processes.
• The findings highlight the need to clarify the mechanisms underlying heat adaptation to guide interventions protecting sleep and occupational safety in a warming world.

## 1 Introduction

Rising global temperatures are negatively influencing a wide range of health outcomes [1,2]. Sleep disruption is one of the hypothesized pathways connecting higher temperatures to several health issues [[3], [4], [5]]. Elevated temperatures increase sleep disturbances, which in turn can lead to a cascade of negative effects for the cardiovascular, metabolic, immune, and cognitive systems [[5], [6], [7], [8], [9]]. According to a recent systematic review, both higher outdoor and indoor temperatures are associated with worse sleep outcomes [10]. Two cumulative mechanisms have been proposed to explain the effects of outdoor and indoor temperatures on sleep. Daytime exposure to high outdoor temperatures can lead to thermal strain, cardiovascular stress, and dehydration, which may carry over into the night [2]. Elevated indoor temperatures at night can hinder the body’s ability to dissipate heat and maintain the optimal core temperature necessary for initiating and maintaining restorative sleep [11]. Nowadays, evidence shows that the negative influence of heat on sleep is stronger during the hottest months of the year, in the warmest regions of the world, and among vulnerable populations such as the elderly, individuals with pre-existing health conditions, or those exposed to prolonged extreme temperatures, such as outdoor workers [3,10,12].

Outdoor workers engaged in physically demanding tasks (e.g., those in the agricultural and construction sectors), are at a higher risk of heat-related injuries and illnesses compared to other vulnerable groups, as they are often required to endure extended exposure to high temperatures during their daily shifts, with minimal control over their work environment [13]. One of the key mechanisms driving an increase in temperature-related injuries among outdoor workers is sleep loss [12,[14], [15], [16]]. Restorative sleep is essential for maintaining vigilance, attention, decision-making, and other complex cognitive functions that are critical for carrying out high-risk tasks safely and preventing workplace injuries [12,17]. Therefore, understanding how heat affects sleep is a key step toward improving worker safety.

Beyond the overall negative effect of heat on sleep, recent literature has questioned whether, and how, humans, especially at-risk groups such as outdoor workers, might adapt to heat-related sleep impacts [10]. By adaptation, we refer here to the progressive reduction in the vulnerability or sensitivity to heat on specific health-related outcomes over the course of a hot season [18,19]. In operational terms, this corresponds to a favorable change in the heat–outcome dose–response relationship, potentially arising from the additive or interactive effects of physiological acclimatization, behavioral changes, and environmental adjustments. However, few studies so far have tested heat adaptation processes in the domain of sleep, and, to our knowledge, no studies have been conducted among outdoor workers despite their heightened vulnerability to heat exposure and associated sleep disruptions [10,12]. Beyond sleep outcomes, a meta-analysis by Brown and colleagues reported that physiological indicators could show adaptive changes at the end of the summer compared to the beginning in participants exposed to heat, suggesting that adaptation can occur at the physiological level [20]. However, the majority of studies included in the meta-analysis focused on highly specific populations, such as healthy and trained male firefighters or military personnel, involved subjects within controlled experimental settings, and relied solely on pre- and post-season assessments [20].

To our knowledge, the present study is the first to examine whether adaptation to heat-related sleep effects occurs among outdoor workers continuously monitored over the summer, combining repeated objective and subjective sleep measures with indoor and outdoor temperature assessments [20]. Our main objective was to quantify time-varying associations between heat and sleep as potential indicators of either adaptation or maladaptation across a summer. Time-varying associations refer here to changes over time in the strength of the temperature–sleep relationship, assessed across June, July, and August. First, we quantified the average effects of indoor and outdoor temperatures on sleep outcomes over the whole summer. We hypothesized that higher temperatures would have a detrimental effect on sleep. Second, we expanded the previous models by examining potential time-varying effects of temperature on sleep across months of data collection. Based on the literature on physiological adaptation to heat, we hypothesized a reduction in the effect of heat on sleep outcomes across the summer [20].

## 2 Methods

### 2.1 Study design and participants

The present work included a sample of 13 Spanish outdoor workers from the agricultural and solar panel sectors, recruited via an open call, posted on social media, and sent to the Independent Farmers’ Association’s network. Eligible participants were adults aged 18 years or older who worked predominantly outdoors during the study period and were able and willing to wear the study device, complete repeated assessments on a compatible smartphone, and provide informed consent. Exclusion criteria included physical or mental health conditions, cognitive impairments, or mobility limitations that could substantially confound the association between heat exposure and sleep or compromise adherence to the study protocol. These participants were involved in an intensive longitudinal study between the 1st of June and the 31st of August 2023 in the region of Catalonia, Spain. The study protocol and instruments were reviewed and approved by the Ethics Committee for Research with Medicines of Parc de Salut MAR.

### 2.2 Sleep outcomes

Participants were asked to wear an activity monitor (Withings Steel HR) on their non-dominant wrist every day and night across the whole summer of 2023. The Steel HR is a multi-sensor activity monitor that provides sleep monitoring through a triaxial accelerometer [21]. Validation studies of consumer sleep-tracking technologies have shown that Withings sensors can provide accurate sleep measures comparable to research-grade actigraphy and polysomnography in some populations, supporting their use for long-term sleep monitoring in ecological contexts [[22], [23], [24]]. Total sleep time (TST) and sleep efficiency (SE) were retrieved from the Withings device. TST represents the total amount of time spent sleeping, and SE is equivalent to the percentage of time spent asleep relative to the total time spent in bed, as detected by the watch [25]. TST was automatically processed using the manufacturer´s algorithm, whereas SE was calculated as [(total sleep time / total time in bed) * 100].

Subjective sleep quality was measured via Ecological Momentary Assessment (EMA) delivered on the participants’ smartphones (via the mobile app M-Path [26]) on mornings following working days. We used a single item (i.e., “How was the quality of your sleep last night?”) with response options ranging from 1 (“very bad”) to 10 (“very good”). This single-item measure has been validated against longer, validated sleep questionnaires and minimizes recall biases [27,28]. Missing EMA observations were excluded from the analyses.

### 2.3 Indoor and outdoor temperature outcomes

Outdoor temperature and relative humidity were calculated for the participant’s work geographical location using gridded interpolated data at a spatial resolution of 300 mprovided by the Catalan Meteorological Agency (Meteocat) [29,30]. Participants’ bedroom indoor temperature and relative humidity were monitored over the night using a thermo-hygrometer (EasyLog EL-USB-2, Lascar Electronics, USA) with 15-minute epoch measurements. For each participant, temperature data were aggregated to match the windows of exposure delimited by the sleep time detected by the Withings device (from sleep onset until sleep end).

Based on preliminary analyses to identify the strongest bivariate correlations between several temperature and sleep outcomes, we selected a 3-day moving average of maximum indoor and outdoor temperatures (covering the night of sleep and the two preceding ones) as the main heat exposure variable for this study. This exposure metric, widely used in environmental research, is more likely to capture the full impact of temperature compared to single-day measures while maintaining parsimony in the models [31]. This choice reflects the assumption that the physiological and behavioral consequences of heat are not confined to a single night but may accumulate over successive days through mechanisms such as sleep debt, thermoregulatory strain, and delayed recovery. Equivalent models using single-night temperature exposure are presented in the Supplemental Material (part 5); the similarity of results across specifications indicates that the observed associations are robust to exposure definition, supporting the 3-day average as a valid representation of short-term heat exposure.

### 2.4 Statistical analyses

We used linear mixed models to estimate the overall pattern of association between temperature and sleep (objective 1), with each sleep outcome as the dependent variable and the 3-day moving average of maximum indoor and outdoor temperatures as the independent variable, thus representing a total of six models (i.e., separate models were conducted for indoor and outdoor temperature). We fitted the models including random intercepts for each participant, acknowledging that individuals may differ in their baseline levels of the outcome. A set of initial covariates was a priori selected based on evidence from previous studies. Then, we selected a final set of covariates based on models’ fit and parsimony using the Akaike Information Criterion (AIC) and Bayesian Information Criterion (BIC). Final linear mixed models included the following covariates: lag 1 sleep outcome to account for sleep autocorrelation relative humidity; work schedule (working days, free days); age; sex (female, male); and the daily number of steps as an indication of the workload for a specific day.

In addition, we tested the role of time in the association between temperature and sleep (objective 2). First, we included months in the model (i.e., 3-level: June, July, August) to test whether sleep outcomes varied from June to August. Then, we created an interaction term between the temperature outcomes and months to specifically test time-varying associations. We complemented this with estimated marginal means (EMMs) analyses. EMMs were used to compare the predicted values of the outcome at specific representative temperatures across months, holding other variables constant. Specifically, continuous covariates were held at their sample mean, and categorical covariates were held at their reference category. This method provides an interpretable way to test how predicted health outcomes differ between months at specific temperature levels, as well as to identify temperature thresholds where the differences between months emerge. For the models with month, we contrasted the predicted values at 26 °C indoor and 30 °C outdoor temperature (each representing the average maximum temperature in this study), while for the models with the interaction, we restricted the analyses to values observed in all months for indoor (20−30 °C) and outdoor (25−35° C) temperatures (i.e., values outside these ranges were excluded to avoid extrapolation beyond observed data). Given prior evidence of potential non-linear temperature–sleep relationships [3], we compared linear and Generalized Additive Models (GAMs) in preliminary analyses; although some non-linearity was detected, linear models demonstrated better overall performance based on AIC and marginal R². Therefore, in this article, we focus exclusively on linear models. To further assess the robustness of the associations detected in the present study, we conducted additional sensitivity analyses adjusting our models for daylight duration, jointly modeling indoor and outdoor temperature, and accounting for individual heterogeneity through random-slope and leave-one-participant-out analyses; full results are presented in the Supplemental Material (parts 6–8). All models were based on complete case analyses and performed using R (version 4.5.0) with the packages lme4 and emmeans. The data and code used in the present study are publicly available on OSF (https://osf.io/5jfkh/).

## 3 Results

### 3.1 Descriptive statistics

A total of 13 participants were included in the study, providing 1,047 observations for TST and SE, and 759 observations for subjective sleep quality across 3 months. Most were male (77%), had a median age of 35.0 years (IQR 30.0–36.0), had completed tertiary education (69%), and worked in the agricultural sector (77%). On average, each participant presented 77 observations of TST and SE (range: 48–95) and 58 subjective sleep quality observations (range: 36–86). Mean score of TST and SE was 416.8 min (SD: 84.1; range: 367 – 494) and 95.4 % (SD: 3.84; range: 91.6 - 97.8), respectively. Participants reported an average subjective sleep quality score of 6.16 (SD: 1.84, range: 4.93 - 7.8). Further descriptive statistics, including monthly and whole-summer summaries of temperature and humidity exposure, are available in the Supplemental Materials (part 1).

### 3.2 Associations between temperature and sleep outcomes (objective 1)

Outdoor temperature was negatively associated with the three sleep outcomes, with each 1 °C increase (3-day mean) resulting in a loss of −2.13 min of TST (95% CI: −3.84, −0.42), a decrease of 0.09 percentage-points (95% CI: −0.17, −0.01) of SE, and a reduction of 0.09 (95% CI: −0.14, −0.04) in subjective sleep quality (see Fig. 1). Regarding indoor temperature, a 1 °C increase was significantly associated with a loss of −3.18 min of TST (95% CI: −5.67, −0.70), a decrease of 0.18 percentage-points in SE (95% CI: −0.30, −0.07), and a −0.17-point reduction in subjective sleep quality (95% CI: −0.24, −0.09). Full model outputs are available in the Supplemental Materials (part 2).

**Fig. 1:**
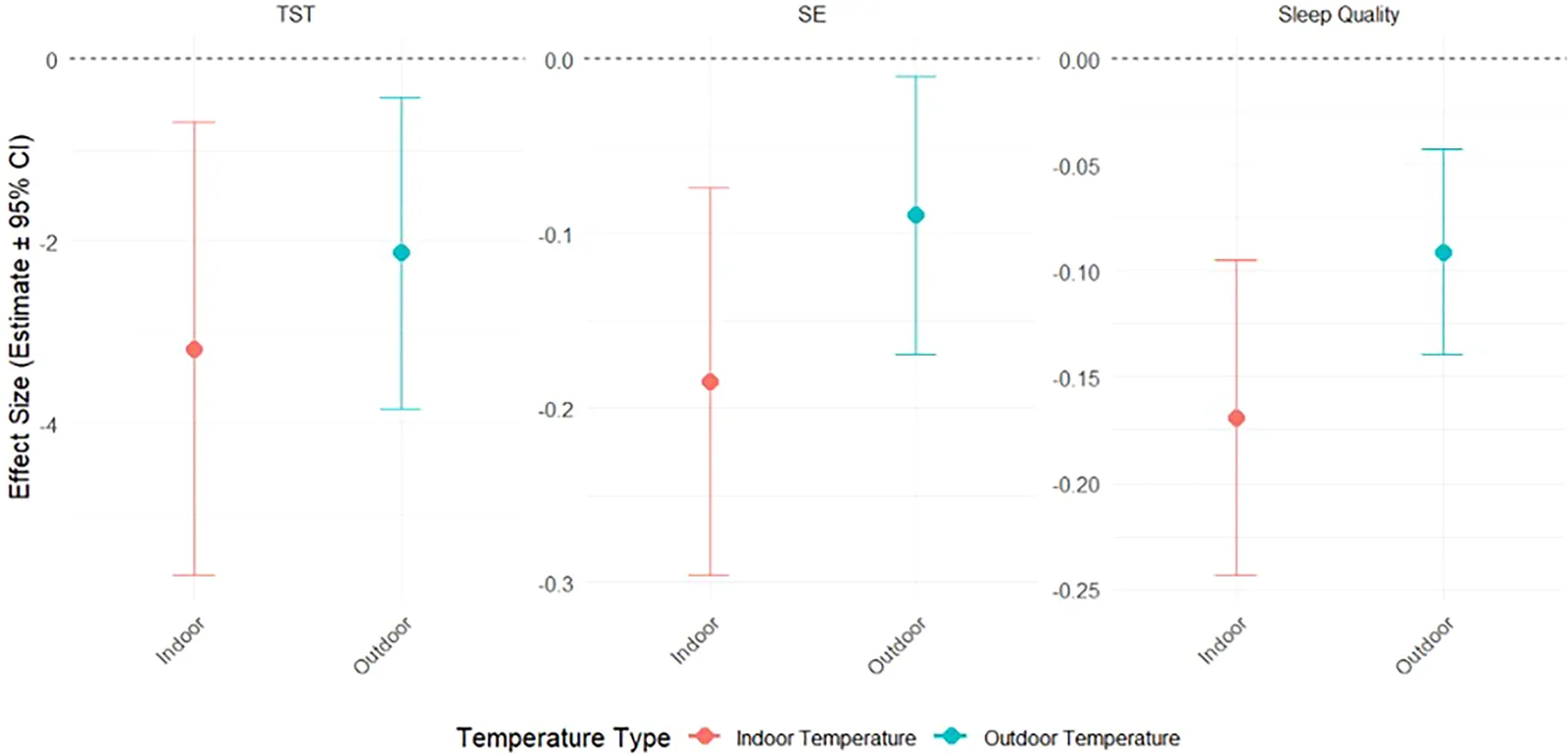
Associations between indoor and outdoor temperatures and sleep outcomes. **Note**. *TST = Total Sleep Time (*min*); SE = Sleep Efficiency (%); Sleep Quality = subjective Sleep Quality (0–10); Effect sizes represent the change in each sleep outcome associated with an average 1* °*C increase across the three days of the 3-day moving average of temperature (*i.e.*, a 1* °*C rise on each of the three days, or an equivalent cumulative increase distributed across them)*.

### 3.3 Time-varying associations between temperature and sleep outcomes (objective 2)

On top of these previous models, we first tested the role of months in the associations between temperature and sleep outcomes. Full models’ outputs are available in Supplemental Materials (part 3). As shown in Fig. 2, we observed an overall pattern of increased TST in August when comparing the predicted values to the previous months at similar indoor and outdoor temperatures (an increase of 24–28 min of sleep in August compared to June and July for comparable temperatures; Fig. 2, panels C and D). In the same vein, subjective sleep quality was higher in August compared to June (but not July) at comparable average outdoor temperature (+0.6 points higher; Fig. 2, panel B).

**Fig. 2:**
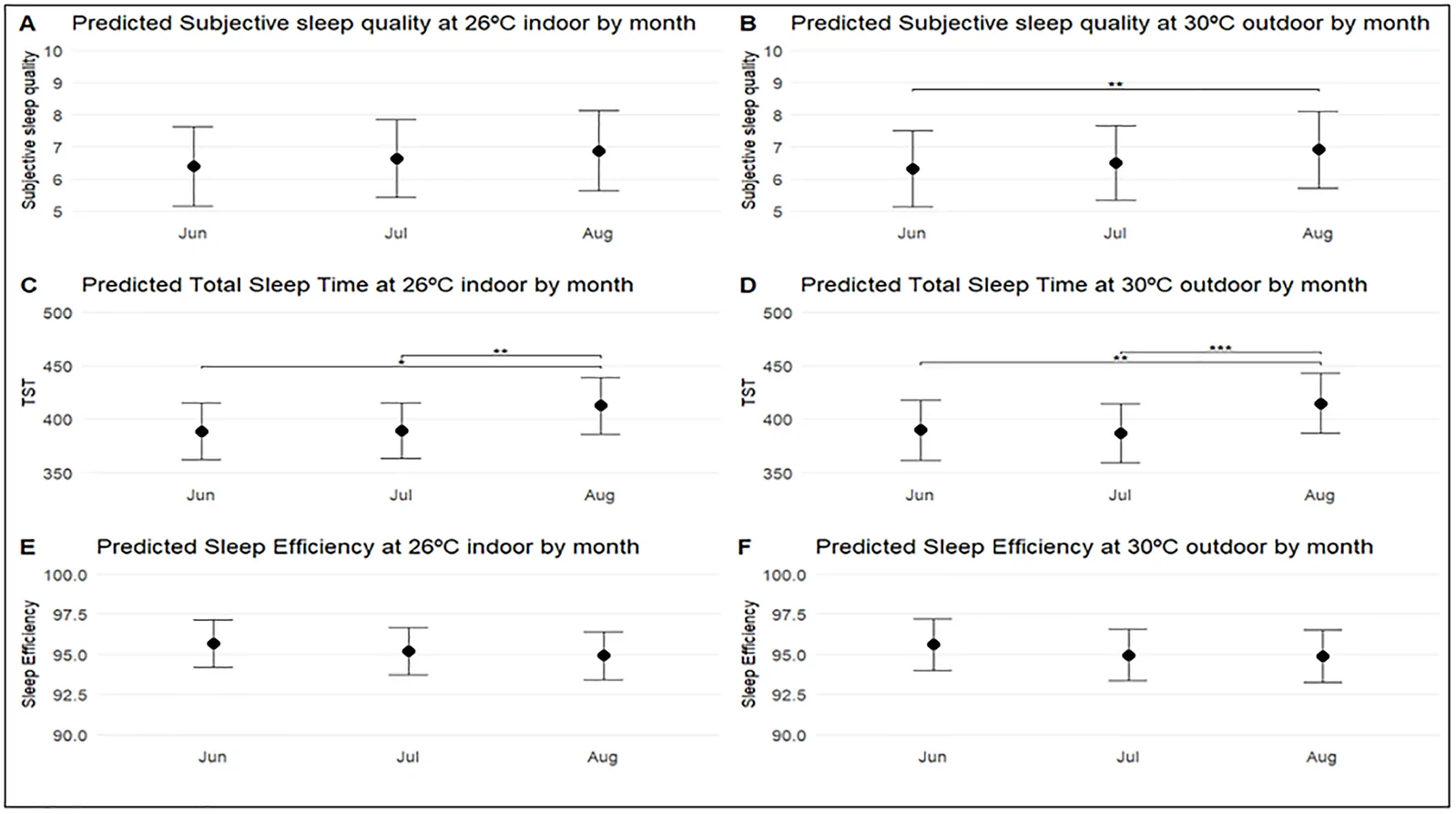
Between-month differences in sleep for fixed temperatures. **Note**. *Error bars represent 95% confidence intervals. Asterisks indicate statistically heterogeneous effect estimates (p < 0.05). Panels A–B show predicted subjective sleep quality, panels C–D total sleep time (TST), and panels E–F sleep efficiency (SE). Predictions were obtained from mixed-effects models including temperature and month as independent terms, with temperature fixed at representative values within the observed range (26* °*C for indoor and 30* °*C for outdoor models)*.

In a third iteration, we included interaction terms between temperature and months in the models. Full models’ outputs are reported in the Supplemental Materials (part 4). Interactions were only significant for subjective sleep quality, for both indoor and outdoor temperatures. Regarding outdoor temperatures, model-based contrasts showed better subjective sleep quality in August compared to June and July, with the largest differences observed around 25–31 °C; beyond this range, the confidence intervals included zero, indicating no significant difference (Fig. 3, panel A). Regarding indoor temperatures, we observed higher predicted subjective sleep quality in August compared to June (Fig. 3, panel B), particularly for temperatures between 22 and 26 °C, with differences narrowing at higher temperatures.

**Fig. 3:**
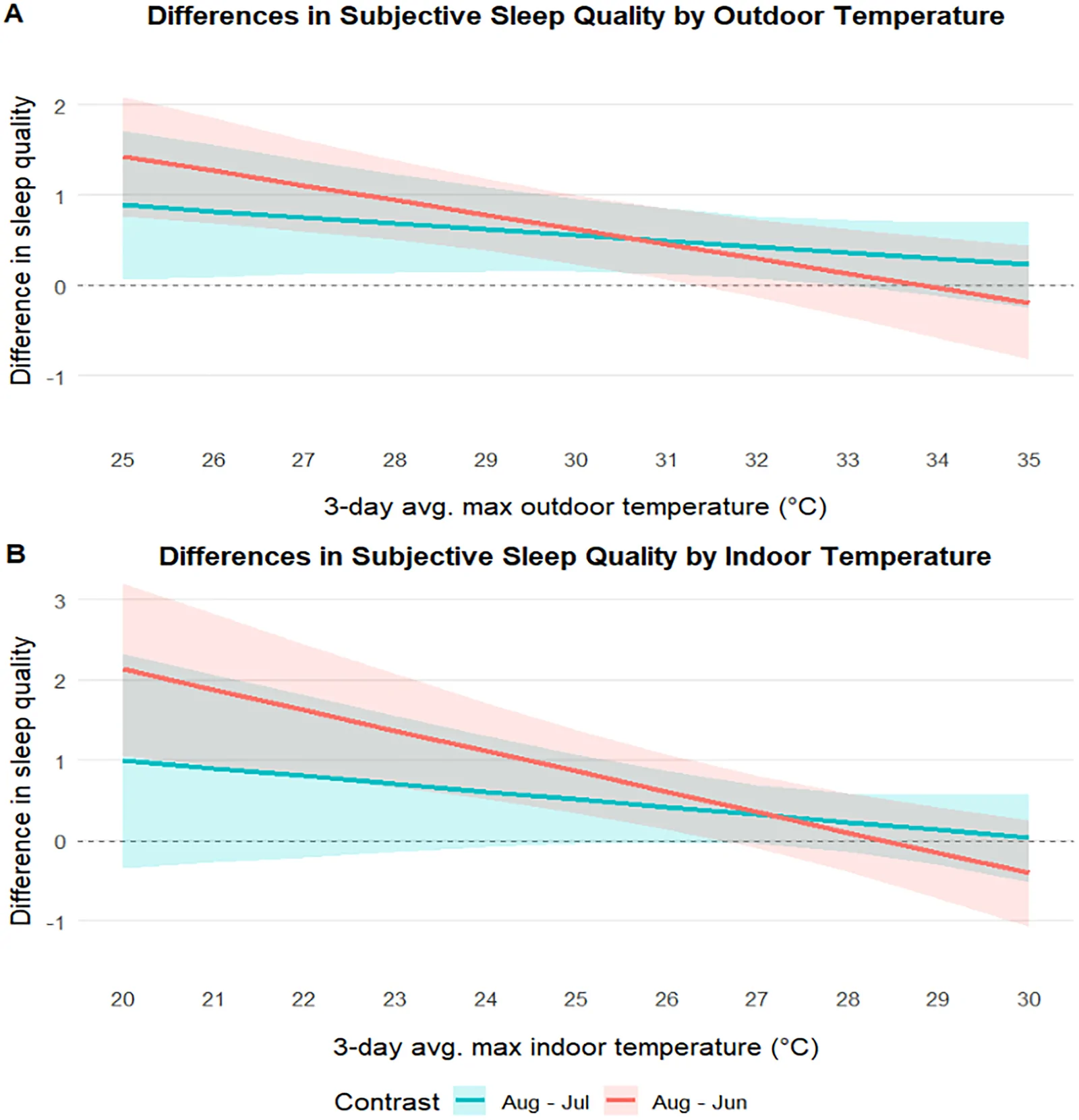
Interaction between temperature and months for outdoor (A) and indoor (B) temperatures. **Note.***Panel A shows the modeled contrasts in predicted subjective sleep quality between August and June (red) and between August and July (blue) across the 3-day average maximum outdoor temperature range. Panel B displays the same contrasts for indoor temperature. Shaded areas represent 95 % confidence intervals. Positive values indicate higher predicted subjective sleep quality in August compared to the reference month. Contrasts were derived from mixed-effects models including an interaction term between temperature (continuous) and month, assuming linear associations within each month. The differences were most evident at moderate temperatures (around 30–32* °*C outdoors and 22–26* °*C indoors), decreasing at higher temperatures where confidence intervals overlapped zero*.

## 4 Discussion

### 4.1 Averaged and time-varying effects of temperature

This study examined the impact of heat on sleep among outdoor workers over the course of a summer, with the primary aim of assessing time-varying associations between heat and sleep as potential indicators of heat adaptation. When considering the average impact of heat on sleep across the whole summer (objective 1), both indoor and outdoor temperatures were found to negatively impact sleep outcomes in concordance with previous literature [10,32]. Indoor temperatures were associated with greater reductions in TST, SE, and subjective sleep quality than outdoor temperatures, but the associations with outdoor temperatures were estimated with greater statistical precision, as reflected in the narrower confidence intervals. The stronger impact of indoor temperature (as compared to outdoors) aligns with previous findings [33,34].

Across the observed interquartile range, these associations corresponded to reductions of 8.86 min in TST, 0.37 percentage points in SE, and 0.38 points in subjective sleep quality for outdoor temperature, and 10.60 min, 0.62 percentage points, and 0.56 points, respectively, for indoor temperature. Although established thresholds for clinically meaningful change are not available for these outcomes, repeated losses of this magnitude across consecutive hot nights are likely relevant for individual functioning and, when experienced across large exposed populations, may translate into meaningful public health and occupational consequences.

We found that sleep was less affected by heat in August than in June and July at comparable temperatures, except for sleep efficiency. We also observed a statistically significant interaction between temperature and time for subjective sleep quality, indicating that within similar temperature ranges and up to certain thresholds, the negative impact of heat on sleep was reduced in August compared to June and July. This pattern could reflect partial adaptation to sustained heat exposure. However, given the small sample size and uncertainty in the estimates, it may also arise from other seasonal changes or unmeasured confounding factors (e.g., summer holidays, changes in work routines or cooling practices). These associations were robust to adjustment for daylight duration (Supplemental Material, part 6), indicating that the observed within-summer attenuation was not explained by seasonal changes in photoperiod alone. Individual-level heterogeneity analyses further indicated that the direction of these associations was consistent across nearly all 13 participants (Supplemental Material, part 8). The attenuation of temperature effects appeared to “plateau” at approximately 30–32 °C outdoors and 26 °C indoors, which may reflect the upper limits of thermoregulatory compensation during sleep under real-world conditions [3]. As climate warming increases the frequency of nights exceeding such thresholds, heat-related sleep disruption may become increasingly difficult to mitigate through “naturally occurring” adaptation.

The outcome-specific pattern may reflect different forms of adaptation. The attenuation in subjective sleep quality may reflect perceptual adaptation, such as reduced sensitivity to heat-related discomfort or altered expectations, as well as behavioral adjustments that improve thermal comfort without necessarily changing sleep continuity. In contrast, the absence of attenuation in sleep efficiency may partly reflect its greater within-person stability, as indicated by its higher intraclass correlation and stronger dependence on stable individual characteristics. Overall, the findings do not support a uniform adaptation response across all the sleep dimensions assessed.

Importantly, the within-summer attenuation observed in this study should not be interpreted as solid evidence of adaptation to long-term climate warming. The study captured changes within a single summer and cannot determine whether any behavioral or physiological adjustments would persist across years, remain effective during prolonged heat exposure, or protect sleep during increasingly frequent and intense heat events. Moreover, temperature remained negatively associated with sleep across the summer, indicating that any possible adjustment did not eliminate heat-related sleep disruption. To our knowledge, however, this is the first study to explicitly address the question of adaptation to heat in relation to sleep using fine-grained continuous monitoring and time-varying associations. While our findings suggest possible time-varying effects consistent with adaptation, this study cannot determine the underlying drivers, whether they stem from behavioral adjustments (e.g., changes in cooling practices or sleep routines), physiological acclimatization (e.g., increased heat tolerance over time), unmeasured shifts in the environmental context (e.g., access to air conditioning or other cooling resources), or a combination of these. Clarifying these mechanisms while replicating our findings would provide a clearer understanding of how adaptation to heat may manifest in sleep outcomes and rule out potential uncontrolled biases. Although we used statistical methods to control for this effect, it is possible that the apparent adaptation pattern may still have been influenced by the higher overall temperature in July compared to August.

### 4.2 Limitations

This study is the first to quantify adaptation to heat in relation to sleep using time-varying associations, and contributes to this literature by using repeated measurements over the summer rather than pre- and post-measures of physiological or behavioral parameters before and after one summer [20]. Other strengths include (i) the extensive behavioral time series collected by our continuous monitoring protocol (averaging 72.85 days and nights per participant, significantly longer than most previous research on temperature and sleep associations); (ii) the inclusion of both objective and subjective measures of sleep, providing a broad understanding of how temperature affects different aspects of sleep; and (iii) the examination of both indoor and outdoor temperatures, addressing a notable gap in prior research and the inclusion of outdoor workers, who are particularly vulnerable to heat [10].

Despite these strengths, the study also has several limitations. First, despite the large number of repeated observations, the participant-level sample was small and consisted primarily of young adult male agricultural workers in Spain. The findings should therefore be considered exploratory and hypothesis-generating and may not generalize to other occupations, demographic groups, or climatic contexts. External validity may have been further limited by recruitment through social media and agricultural networks, which may have introduced self-selection bias by preferentially attracting more motivated or health-conscious individuals.

Second, sleep was assessed using the Withings Steel HR, a consumer-grade accelerometer relying on a proprietary algorithm that has not been specifically validated in heat-exposed outdoor workers. Accelerometer-based devices may misclassify quiet wakefulness as sleep and overestimate sleep efficiency, potentially during hot nights when participants remain awake but inactive. The device did not provide sleep-stage information and did not detect sleep episodes shorter than three hours. Consequently, short daytime naps used to compensate for nocturnal sleep loss may have been missed, leading to an underestimation of total daily sleep. Subjective sleep quality was also available for fewer nights because it depended on active EMA completion, and missing responses may not have occurred randomly.

Third, outdoor temperature was derived from gridded meteorological data rather than direct worksite measurements, potentially introducing exposure misclassification and limiting the characterization of occupational heat stress in the absence of on-site wet-bulb globe temperature measurements. Indoor temperature also reflected housing characteristics, ventilation, air-conditioning use, and personal cooling practices, which were not systematically assessed.

Finally, because participants were monitored during a single summer, the month variable could capture only short-term temporal changes and served as an indirect indicator of adaptation. While the weaker temperature–sleep associations observed in August are compatible with partial adaptation, alternative explanations cannot be excluded. Residual confounding from unmeasured factors (e.g., air-conditioning use during warm nights) remains possible. Ceiling effects and sparse observations at the highest temperatures may also have contributed to the apparent attenuation. Moreover, the three-day temperature average captured only short-term heat exposure, while the limited length and completeness of the time series precluded reliable assessment of longer lag windows or distributed lag models. Larger studies spanning multiple summers, with more diverse workers, direct occupational heat measurements, and detailed information on cooling and work practices, are needed to confirm these findings.

### 4.3 Conclusion

This study is, to our knowledge, the first to explicitly investigate potential adaptation to heat in relation to sleep by testing time-varying associations across a summer. Consistent with previous research, we found that both indoor and outdoor temperatures were associated with poorer sleep outcomes. Beyond these average effects, our analyses revealed patterns suggesting reduced heat-related impacts on sleep later in the summer, only for subjective sleep quality, and up to identifiable temperature thresholds. While these findings are consistent with a process of adaptation, our study cannot determine the underlying mechanisms, which may involve behavioral, psychophysiological, or contextual changes. Clarifying these drivers and testing whether similar patterns emerge in more diverse populations, under different climatic conditions, and with larger sample sizes will be essential to understanding how humans adjust to heat in ways that preserve sleep. Such insights could inform targeted interventions to protect sleep health and, ultimately, occupational safety in a warming world.

## Funding statement

We acknowledge support from the Spanish Ministry of Science and Innovation and State Research Agency through the “Centro de Excelencia Severo Ochoa 2019–2023″ Program (CEX2018-000806-S) funded by MCIN/AEI/ 10.13039/501100011033, and support from the Generalitat de Catalunya through the CERCA Program. GC has also been awarded with the grant RYC2021-033537-I, supported by MCIN/AEI/10.13039/501100011033 and by the European Union "NextGenerationEU"/PRTR”; and from the 10.13039/501100003407Ministry of Research and Universities of the Government of Catalonia (2021 SGR 01563).

## CRediT authorship contribution statement

**Andrea Montanari:** Writing – original draft, Methodology, Investigation, Formal analysis, Data curation, Conceptualization. **Constanza Vielma:** Writing – review & editing, Visualization, Investigation, Formal analysis, Data curation. **Joan Ballester:** Writing – review & editing. **Xavier Basagaña:** Writing – review & editing. **Tarik Benmarhnia:** Writing – review & editing, Methodology. **Basile Chaix:** Writing – review & editing. **Guillaume Chevance:** Writing – review & editing, Supervision, Methodology, Investigation, Funding acquisition, Conceptualization.

## Declaration of competing interest

The authors declare that they have no known competing financial interests or personal relationships that could have appeared to influence the work reported in this paper.

## Footnotes

Supplementary material associated with this article can be found, in the online version, at doi:10.1016/j.joclim.2026.100747.
