# Supplementary material for: Time-varying associations of temperature and sleep as a marker of heat adaptation: A case study of Spanish outdoor workers over one summer of continuous monitoring
Source: J Clim Chang Health. 2026 Oct 17;32:100747. doi: 10.1016/j.joclim.2026.100747 (PMC13691138; doi:10.1016/j.joclim.2026.100747)
Supplement: Supplementary file 1 [file mmc1.docx]

**Time-varying associations of temperature and sleep as a marker of heat adaptation: a case study of Spanish outdoor workers over one summer of continuous monitoring**

**SUPPLEMENTAL MATERIAL**

**INDEX**

[**Main Supplemental Material**	2](#_Toc235715925)

[1.	Additional descriptive statistics	2](#_Toc235715926)

[2.	Main models (objective 1)	9](#_Toc235715927)

[3.	Models with months (objective 2)	12](#_Toc235715928)

[4.	Models with interactions (objective 2)	15](#_Toc235715929)

[**Sensitivity Analysis (Robustness check)**	18](#_Toc235715930)

[5.	Models with single day temperatures	18](#_Toc235715931)

[6.	Models adjusted for daylight duration	26](#_Toc235715932)

[7.	Models with both temperatures combined	29](#_Toc235715933)

[8.	Models accounting for individual heterogeneity	31](#_Toc235715934)

[Random slope models	31](#_Toc235715935)

[Leave-One-Out analysis (LOO)	35](#_Toc235715936)

# **Main Supplemental Material**

## 1. Additional descriptive statistics

Table 1 summarizes the descriptive statistics for sleep outcomes across all participants, including total sleep time (TST), sleep efficiency (SE), and Subjective Sleep Quality assessed by ecological momentary assessment (EMA). The table highlights individual variability in sleep patterns and the proportion of missing data for each measure.

**Table 1**

*Sleep Outcomes Descriptive Statistics*

| **Participant Level** | **TST  m (SD)** | **SE  m (SD)** | **TST SE  N (NA, NA%)** | **Sleep Quality  m (SD)** | **Sleep Quality  N (NA, NA%)** |
| --- | --- | --- | --- | --- | --- |
| **1** | 373 (65.7) | 94.8 (4.54) | 95 (2, 2.1%) | 4.93 (1.77) | 68 (29, 29.9%) |
| **2** | 399 (94.7) | 92.6 (4.22) | 91 (5, 5.2%) | 6.29 (1.24) | 70 (26, 27.1%) |
| **3** | 443 (69.4) | 97.1 (2.61) | 91 (6, 6.2%) | 5.36 (0.83) | 45 (52, 53.6%) |
| **4** | 408 (74.4) | 95.4 (2.86) | 89 (7, 7.3%) | 7.8 (0.96) | 75 (21, 21.9%) |
| **5** | 419 (72.9) | 97.8 (2.65) | 83 (14, 14.4%) | 6.97 (1.04) | 64 (33, 34.0%) |
| **6** | 390 (76.2) | 96.2 (3.04) | 84 (13, 13.4%) | 5.42 (2.56) | 55 (42, 43.3%) |
| **7** | 405 (74.8) | 93.5 (3.61) | 88 (6, 6.4%) | 6.31 (1.85) | 51 (43, 45.7%) |
| **8** | 367 (109) | 91.6 (5.27) | 48 (46, 48.9%) | 7.39 (1.5) | 38 (56, 59.6%) |
| **9** | 494 (77.3) | 97.2 (2.24) | 91 (4, 4.2%) | 6.57 (1.53) | 86 (9, 9.5%) |
| **10** | 405 (75.3) | 97.1 (2.50) | 76 (21, 21.6%) | 5.28 (2.21) | 57 (40, 41.2%) |
| **11** | 434 (66.8) | 95.9 (4.03) | 79 (16, 16.8%) | 6.04 (1.40) | 55 (40, 42.1%) |
| **12** | 447 (70.3) | 94.2 (2.76) | 59 (36, 37.9%) | 5.71 (1.59) | 59 (36, 37.9%) |
| **13** | 425 (90.5) | 94.6 (3.64) | 73 (24, 24.7%) | 5.28 (2.26) | 36 (61, 62.9%) |
| **Group Level** | 416.8 (84.1) | 95.4 (3.84) | 1047 (200, 16%) | 6.16 (1.84) | 759 (488, 39.1%) |

*m: mean; SD: standard deviation, N: number of observations; NA: number of missing observations*

Table 2 presents the median and standard deviation of sleep outcomes by month, highlighting temporal variations in TST, SE, and Sleep Quality scores. Notably, TST and Sleep Quality scores tended to increase during the month of August, suggesting that participants experienced longer and better-perceived sleep towards the end of the summer.

***Table 2***

*Sleep Outcomes Descriptive Statistics by Month*

| **Month^1^** | **TST** | |  | **SE** | |  | **Sleep Quality** | |  |
| --- | --- | --- | --- | --- | --- | --- | --- | --- | --- |
|  | *Median* | *SD* | *N(NA%)* | *Median* | *SD* | *N(NA)* | *Median* | *SD* | *N(NA)* |
| ***June*** | 415 | 82.34 | 351 (10%) | 96.59 | 3.49 | 349 (10.5%) | 6 | 1.77 | 267 (31.5%) |
| ***July*** | 407 | 81.68 | 349 (13.4%) | 96.36 | 4.13 | 344 (85.4%) | 6 | 1.85 | 265 (34.2%) |
| ***August*** | 435 | 87.01 | 321 (20%) | 95.85 | 3.93 | 316 (11.2%) | 7 | 1.9 | 226 (43.6%) |

*Median and Standard Deviation (SD) of Minutes of Total Sleep Time (TST), Percentage of Sleep Efficiency (SE) and Subjective Score (range: 1-10) of Sleep assessed by Ecological Momentary Assessment (Sleep Quality), by Month. Number of observations: N^1^ = 891 N^2^=918*

Table 3 provides the descriptive statistics for maximum indoor and outdoor temperatures by month, detailing daily means, standard deviations, and three-day moving averages.

***Table 3***

*Temperature Descriptive Statistics by Month*

| **Month** | **Max Outdoor Temperature** | | | | **Max Indoor Temperature** | | | |
| --- | --- | --- | --- | --- | --- | --- | --- | --- |
|  | **Daily Mean** | **Daily  SD** | **3-day  MA Mean** | **3-day  MA SD** | **Daily Mean** | **Daily SD** | **3-day MA Mean** | **3-day MA SD** |
| *June* | 28.97 | 3.02 | 29.06 | 2.89 | 24.50 | 2.70 | 24.67 | 2.67 |
| *July* | 31.37 | 2.69 | 31.26 | 2.31 | 27.50 | 1.85 | 27.43 | 1.80 |
| *August* | 32.03 | 4.02 | 31.86 | 3.46 | 27.50 | 2.39 | 27.56 | 2.21 |

*Median and Standard Deviation (SD) of Daily and 3-day Moving Average (MA) of Max Temperature Indoor and Outdoor by Month*

Figure 1 illustrates the fluctuations in temperature over the whole summer and by month.

**Figure 1**

*Temperature across Time.*

|  |  |
| --- | --- |

Figure 2 presents overall, monthly, and participant-level distributions of observations across indoor and outdoor temperature strata and above versus below key temperature thresholds.

**Figure 2.**

*Observations by temperature stratum*

|  |  |
| --- | --- |

|  |  |
| --- | --- |

## 2. Main models (objective 1)

**Table 4a**.

*Linear Models of Outdoor Temperature and Sleep Outcomes.*

|  | **TST** | | | **SE** | | | **Sleep Quality** | | |
| --- | --- | --- | --- | --- | --- | --- | --- | --- | --- |
| *Predictors* | *Est.* | *CI* | *p* | *Est.* | *CI* | *p* | *Est.* | *CI* | *p* |
| (Intercept) | 347.50 | 138.68, 556.32 | **0.001** | 96.16 | 82.77, 109.55 | **<0.001** | 10.31 | 5.60, 15.01 | **<0.001** |
| Lagged Outcome | 0.02 | -0.05, 0.09 | 0.565 | 0.02 | -0.05, 0.09 | 0.599 | 0.22 | 0.14, 0.30 | **<0.001** |
| Workday [1] | 1.72 | -12.15, 15.60 | 0.807 | 0.73 | 0.08, 1.38 | **0.028** | -0.23 | -2.42, 1.96 | 0.837 |
| Age | 2.72 | -1.47, 6.92 | 0.203 | 0.05 | -0.19, 0.29 | 0.667 | -0.03 | -0.10, 0.04 | 0.428 |
| Sex | 6.75 | -42.38, 55.88 | 0.787 | -0.60 | -3.43, 2.23 | 0.677 | -0.84 | -1.71, 0.03 | 0.058 |
| Max Outdoor T 3day MA | -2.13 | -3.84, -0.42 | **0.015** | -0.09 | -0.17, -0.01 | **0.027** | -0.09 | -0.14, -0.04 | **<0.001** |
| Steps (thousands) | -1.98 | -3.36, -0.60 | **0.005** | -0.04 | -0.11, 0.03 | 0.237 | -0.02 | -0.07, 0.02 | 0.260 |
| Outdoor Humidity | 0.28 | -0.33, 0.89 | 0.361 | -0.01 | -0.04, 0.02 | 0.390 | 0.00 | -0.01, 0.02 | 0.726 |
| **Additional Information** | | | | | | | | | |
| σ^2^ | 5313.99 | | | 11.26 | | | 2.37 | | |
| τ_00_ | 1199.57 _participant_ | | | 4.10 _participant_ | | | 0.33 _participant_ | | |
| ICC | 0.18 | | | 0.27 | | | 0.12 | | |
| N | 13 _participant_ | | | 13 _participant_ | | | 13 _participant_ | | |
| Observations | 880 | | | 867 | | | 529 | | |
| Marginal R^2^ / Conditional R^2^ | 0.041 / 0.218 | | | 0.021 / 0.283 | | | 0.171 / 0.272 | | |

*Estimates = Effect size; CI (Confidence Interval) = Range of true effect; σ² (Residual Variance) = Unexplained variability; τ00 (Intercept Variance) = Group variability; ICC (Intraclass Correlation Coefficient) = Group effect proportion; Marginal R² = Fixed effects variance; Conditional R² = Total variance (fixed + random).*

**Table 4b.**

*Linear Models of Indoor Temperature and Sleep Outcomes*

|  |  | | **TST** | | | **SE** | | | **Sleep Quality** | | |
| --- | --- | --- | --- | --- | --- | --- | --- | --- | --- | --- | --- |
| *Predictors* |  | | *Est.* | *CI* | *p* | *Est.* | *CI* | *p* | *Est.* | *CI* | *p* |
| (Intercept) |  | | 442.28 | 226.88, 657.68 | **<0.001** | 100.92 | 88.21, 113.63 | **<0.001** | 15.04 | 9.27, 20.80 | **<0.001** |
| Lagged Outcome |  | | 0.02 | -0.05, 0.08 | 0.583 | 0.01 | -0.05, 0.07 | 0.721 | 0.22 | 0.14, 0.30 | **<0.001** |
| Workday [1] |  | | -1.49 | -14.20, 11.22 | 0.818 | 0.60 | 0.02, 1.18 | **0.044** | -0.41 | -2.60, 1.79 | 0.716 |
| Age |  | | 2.42 | -1.53, 6.36 | 0.229 | 0.03 | -0.18, 0.25 | 0.778 | -0.04 | -0.13, 0.05 | 0.385 |
| Sex |  | | 8.64 | -37.29, 54.56 | 0.712 | -0.56 | -3.07, 1.96 | 0.664 | -0.63 | -1.68, 0.42 | 0.236 |
| Max Indoor T 3day MA |  | | -3.18 | -5.67, -0.70 | **0.012** | -0.18 | -0.30, -0.07 | **0.001** | -0.17 | -0.24, -0.09 | **<0.001** |
| Steps (thousands) |  | | -2.22 | -3.58, -0.86 | **0.001** | -0.03 | -0.09, 0.03 | 0.365 | -0.02 | -0.06, 0.02 | 0.383 |
| Indoor Humidity |  | | -0.71 | -1.86, 0.44 | 0.223 | -0.03 | -0.09, 0.02 | 0.188 | -0.05 | -0.08, -0.01 | **0.004** |
| **Additional Information** | |  | | | | | | | | | |
| σ^2^ |  | | 5627.01 | | | 11.38 | | | 2.38 | | |
| τ_00_ |  | | 1050.19 _participant_ | | | 3.23 _participant_ | | | 0.51 _participant_ | | |
| ICC |  | | 0.16 | | | 0.22 | | | 0.18 | | |
| N |  | | 13 _participant_ | | | 13 _participant_ | | | 13 _participant_ | | |
| Observations |  | | 963 | | | 956 | | | 546 | | |
| Marginal R^2^ / Conditional R^2^ |  | | 0.035 / 0.187 | | | 0.029 / 0.244 | | | 0.157 / 0.307 | | |

*Estimates = Effect size; CI (Confidence Interval) = Range of true effect; σ² (Residual Variance) = Unexplained variability; τ00 (Intercept Variance) = Group variability; ICC (Intraclass Correlation Coefficient) = Group effect proportion; Marginal R² = Fixed effects variance; Conditional R² = Total variance (fixed + random).*

## 3. Models with months (objective 2)

**Table 5a**.

*Linear Models of Outdoor Temperature and Sleep Outcomes by Month.*

|  | **TST** | | | **SE** | | | **Sleep Quality** | | |
| --- | --- | --- | --- | --- | --- | --- | --- | --- | --- |
| *Predictors* | *Est.* | *CI* | *p* | *Est.* | *CI* | *p* | *Est.* | *CI* | *p* |
| (Intercept) | 356.15 | 145.07, 567.23 | **0.001** | 96.56 | 82.87, 110.24 | **<0.001** | 10.73 | 6.07, 15.39 | **<0.001** |
| Lagged Outcome | -0.01 | -0.07, 0.06 | 0.836 | 0.00 | -0.07, 0.07 | 0.953 | 0.20 | 0.12, 0.29 | **<0.001** |
| Workday | 1.24 | -12.65, 15.13 | 0.861 | 0.79 | 0.13, 1.45 | **0.019** | -0.27 | -2.46, 1.91 | 0.806 |
| Age | 2.42 | -1.82, 6.65 | 0.263 | 0.06 | -0.19, 0.30 | 0.655 | -0.04 | -0.11, 0.04 | 0.337 |
| Sex | 6.70 | -42.92, 56.32 | 0.791 | -0.64 | -3.54, 2.26 | 0.663 | -0.84 | -1.70, 0.01 | 0.053 |
| Month [Jul] | -2.94 | -15.66, 9.77 | 0.650 | -0.66 | -1.26, -0.06 | **0.030** | 0.20 | -0.14, 0.54 | 0.255 |
| Month [Aug] | 25.12 | 11.28, 38.96 | **<0.001** | -0.71 | -1.36, -0.06 | **0.033** | 0.60 | 0.23, 0.97 | **0.002** |
| Max Outdoor T 3day MA | -2.60 | -4.50, -0.71 | **0.007** | -0.04 | -0.13, 0.05 | 0.381 | -0.11 | -0.17, -0.06 | **<0.001** |
| Steps (thousands) | -1.79 | -3.17, -0.41 | **0.011** | -0.05 | -0.12, 0.02 | 0.135 | -0.02 | -0.06, 0.02 | 0.318 |
| Outdoor Humidity | 0.57 | -0.05, 1.18 | 0.072 | -0.01 | -0.04, 0.02 | 0.426 | 0.01 | -0.01, 0.02 | 0.364 |
| **Additional Information** | | | | | | | | | |
| σ^2^ | 5204.73 | | | 11.30 | | | 2.34 | | |
| τ_00_ | 1225.97 _participant_ | | | 4.30 _participant_ | | | 0.32 _participant_ | | |
| ICC | 0.19 | | | 0.28 | | | 0.12 | | |
| N | 13 _participant_ | | | 13 _participant_ | | | 13 _participant_ | | |
| Observations | 859 | | | 840 | | | 528 | | |
| Marginal R^2^ / Conditional R^2^ | 0.058 / 0.238 | | | 0.026 / 0.295 | | | 0.189 / 0.286 | | |

*Estimates = Effect size; CI (Confidence Interval) = Range of true effect; σ² (Residual Variance) = Unexplained variability; τ00 (Intercept Variance) = Group variability; ICC (Intraclass Correlation Coefficient) = Group effect proportion; Marginal R² = Fixed effects variance; Conditional R² = Total variance (fixed + random).*

**Table 5b**.

*Linear Models of Indoor Temperature and Sleep Outcomes by Month.*

|  | **TST** | | | **SE** | | | **Sleep Quality** | | |
| --- | --- | --- | --- | --- | --- | --- | --- | --- | --- |
| *Predictors* | *Est.* | *CI* | *p* | *Est.* | *CI* | *p* | *Est.* | *CI* | *p* |
| (Intercept) | 404.99 | 186.86, 623.12 | **<0.001** | 102.13 | 88.80, 115.47 | **<0.001** | 14.72 | 8.82, 20.62 | **<0.001** |
| Lagged Outcome | -0.01 | -0.07, 0.06 | 0.857 | -0.00 | -0.06, 0.06 | 0.982 | 0.21 | 0.13, 0.29 | **<0.001** |
| Workday [1] | -0.46 | -13.21, 12.30 | 0.944 | 0.65 | 0.06, 1.24 | **0.032** | -0.45 | -2.65, 1.75 | 0.688 |
| Age | 2.19 | -1.77, 6.14 | 0.278 | 0.04 | -0.19, 0.27 | 0.728 | -0.04 | -0.14, 0.05 | 0.354 |
| Sex | 9.97 | -36.11, 56.04 | 0.671 | -0.61 | -3.25, 2.02 | 0.649 | -0.62 | -1.71, 0.47 | 0.266 |
| Month [Jul] | 0.58 | -13.52, 14.69 | 0.935 | -0.44 | -1.09, 0.20 | 0.176 | 0.25 | -0.13, 0.62 | 0.198 |
| Month [Aug] | 24.26 | 7.62, 40.89 | **0.004** | -0.74 | -1.50, 0.02 | 0.056 | 0.49 | 0.04, 0.93 | **0.033** |
| Max Indoor T 3day MA | -3.91 | -6.80, -1.01 | **0.008** | -0.13 | -0.26, 0.00 | 0.054 | -0.20 | -0.28, -0.12 | **<0.001** |
| Steps (thousands) | -2.26 | -3.63, -0.90 | **0.001** | -0.04 | -0.10, 0.03 | 0.263 | -0.02 | -0.06, 0.02 | 0.422 |
| Indoor Humidity | 0.34 | -0.95, 1.63 | 0.602 | -0.06 | -0.12, 0.00 | 0.061 | -0.03 | -0.06, 0.01 | 0.114 |
| **Additional Information** | | | | | | | | | |
| σ^2^ | 5550.47 | | | 11.46 | | | 2.37 | | |
| τ_00_ | 1054.64 _participant_ | | | 3.56 _participant_ | | | 0.56 _participant_ | | |
| ICC | 0.16 | | | 0.24 | | | 0.19 | | |
| N | 13 _participant_ | | | 13 _participant_ | | | 13 _participant_ | | |
| Observations | 939 | | | 924 | | | 545 | | |
| Marginal R^2^ / Conditional R^2^ | 0.047 / 0.199 | | | 0.029 / 0.259 | | | 0.165 / 0.324 | | |

*Estimates = Effect size; CI (Confidence Interval) = Range of true effect; σ² (Residual Variance) = Unexplained variability; τ00 (Intercept Variance) = Group variability; ICC (Intraclass Correlation Coefficient) = Group effect proportion; Marginal R² = Fixed effects variance; Conditional R² = Total variance (fixed + random).*

## 4. Models with interactions (objective 2)

**Table 6a**.

*Linear Models of Outdoor Temperature and Sleep Outcomes including interaction between Temperature and Month.*

|  | **TST** | | | **SE** | | | **Sleep Quality** | | |
| --- | --- | --- | --- | --- | --- | --- | --- | --- | --- |
| *Predictors* | *Est.* | *CI* | *p* | *Est.* | *CI* | *p* | *Est.* | *CI* | *p* |
| (Intercept) | 331.15 | 105.64, 556.66 | **0.004** | 95.61 | 81.42, 109.81 | **<0.001** | 7.96 | 2.92, 13.00 | **0.002** |
| Lagged Outcome | -0.01 | -0.08, 0.06 | 0.785 | 0.00 | -0.07, 0.07 | 0.971 | 0.18 | 0.10, 0.26 | **<0.001** |
| Workday [1] | 1.50 | -12.42, 15.43 | 0.832 | 0.75 | 0.09, 1.41 | **0.026** | -0.31 | -2.48, 1.87 | 0.782 |
| Age | 2.43 | -1.82, 6.68 | 0.262 | 0.05 | -0.19, 0.30 | 0.664 | -0.04 | -0.11, 0.04 | 0.313 |
| Sex | 6.91 | -42.84, 56.66 | 0.785 | -0.64 | -3.54, 2.27 | 0.667 | -0.87 | -1.74, 0.00 | 0.050 |
| Month [Jul] | 72.30 | -62.35, 206.96 | 0.292 | -3.54 | -9.83, 2.74 | 0.269 | 2.94 | -0.60, 6.48 | 0.103 |
| Month [Aug] | 48.21 | -67.61, 164.03 | 0.414 | 1.95 | -3.48, 7.39 | 0.481 | 5.49 | 2.31, 8.67 | **0.001** |
| Max Outdoor T 3day MA | -1.69 | -4.85, 1.47 | 0.294 | -0.01 | -0.16, 0.13 | 0.844 | -0.01 | -0.10, 0.07 | 0.771 |
| Steps (thousands) | -1.86 | -3.26, -0.47 | **0.009** | -0.05 | -0.11, 0.02 | 0.180 | -0.02 | -0.07, 0.02 | 0.240 |
| Outdoor Humidity | 0.56 | -0.07, 1.19 | 0.081 | -0.01 | -0.04, 0.02 | 0.628 | 0.01 | -0.01, 0.03 | 0.218 |
| Month [Jul] × max outdoor T 3day MA | -2.48 | -6.92, 1.96 | 0.273 | 0.09 | -0.12, 0.30 | 0.397 | -0.10 | -0.21, 0.02 | 0.106 |
| Month [Aug] × max outdoor T 3day MA | -0.83 | -4.65, 3.00 | 0.672 | -0.08 | -0.26, 0.10 | 0.356 | -0.16 | -0.27, -0.06 | **0.002** |
| **Additional Information** | | | | | | | | | |
| σ^2^ | 5209.23 | | | 11.29 | | | 2.31 | | |
| τ_00_ | 1232.91 _participant_ | | | 4.31 _participant_ | | | 0.33 _participant_ | | |
| ICC | 0.19 | | | 0.28 | | | 0.13 | | |
| N | 13 _participant_ | | | 13 _participant_ | | | 13 _participant_ | | |
| Observations | 859 | | | 840 | | | 528 | | |
| Marginal R^2^ / Conditional R^2^ | 0.060 / 0.240 | | | 0.028 / 0.297 | | | 0.199 / 0.300 | | |

*Estimates = Effect size; CI (Confidence Interval) = Range of true effect; σ² (Residual Variance) = Unexplained variability; τ00 (Intercept Variance) = Group variability; ICC (Intraclass Correlation Coefficient) = Group effect proportion; Marginal R² = Fixed effects variance; Conditional R² = Total variance (fixed + random). The models include an interaction term between temperature and month to test for time-varying associations.*

**Table 6b**

*Linear Models of Indoor Temperature and Sleep Outcomes including interaction between Temperature and Month.*

|  |  | ***TST*** |  | ***SE*** |  |  | **Sleep Quality** |  |  |
| --- | --- | --- | --- | --- | --- | --- | --- | --- | --- |
| *Predictors* | *Estimates* | *CI* | *p* | *Estimates* | *CI* | *p* | *Estimates* | *CI* | *p* |
| (Intercept) | 433.51 | 205.46, 661.57 | **<0.001** | 102.23 | 88.44, 116.02 | **<0.001** | 12.23 | 6.22, 18.23 | **<0.001** |
| Lagged Outcome | -0.01 | -0.07, 0.06 | 0.810 | -0.00 | -0.07, 0.06 | 0.960 | 0.18 | 0.10, 0.26 | **<0.001** |
| Workday [1] | 0.31 | -12.48, 13.10 | 0.962 | 0.64 | 0.04, 1.23 | **0.036** | -0.39 | -2.57, 1.79 | 0.725 |
| Age | 2.16 | -1.75, 6.06 | 0.278 | 0.04 | -0.19, 0.27 | 0.728 | -0.04 | -0.13, 0.05 | 0.349 |
| Sex | 10.51 | -34.99, 56.01 | 0.650 | -0.63 | -3.28, 2.03 | 0.643 | -0.64 | -1.72, 0.44 | 0.247 |
| Month [Jul] | 71.11 | -76.98, 219.20 | 0.346 | -2.62 | -9.38, 4.14 | 0.447 | 4.30 | 0.31, 8.30 | **0.035** |
| Month [Aug] | -37.97 | -176.61, 100.66 | 0.591 | -0.55 | -6.90, 5.79 | 0.864 | 7.21 | 3.29, 11.14 | **<0.001** |
| Max Indoor T 3day MA | -4.41 | -8.11, -0.71 | **0.020** | -0.14 | -0.31, 0.03 | 0.106 | -0.10 | -0.20, 0.00 | 0.056 |
| Steps (thousands) | -2.35 | -3.72, -0.98 | **0.001** | -0.03 | -0.10, 0.03 | 0.290 | -0.02 | -0.07, 0.02 | 0.274 |
| Indoor Humidity | 0.11 | -1.22, 1.44 | 0.875 | -0.05 | -0.11, 0.01 | 0.088 | -0.02 | -0.06, 0.01 | 0.175 |
| Month [Jul] × Max Indoor  T 3day MA | -2.57 | -8.14, 3.00 | 0.365 | 0.08 | -0.17, 0.34 | 0.530 | -0.16 | -0.31, -0.01 | **0.039** |
| Month [Aug] × Max Indoor  T 3day MA | 2.24 | -2.92, 7.39 | 0.394 | -0.00 | -0.24, 0.23 | 0.969 | -0.25 | -0.40, -0.11 | **0.001** |
| **Additional Information** | | | | | | | | | |
| σ^2^ | 5550.10 | | | 11.48 | | | 2.32 | | |
| τ_00_ | 1026.06 _participant_ | | | 3.60 _participant_ | | | 0.55 _participant_ | | |
| ICC | 0.16 | | | 0.24 | | | 0.19 | | |
| N | 13 _participant_ | | | 13 _participant_ | | | 13 _participant_ | | |
| Observations | 939 | | | 924 | | | 545 | | |
| Marginal R^2^ / Conditional R^2^ | 0.051 / 0.199 | | | 0.029 / 0.261 | | | 0.183 / 0.339 | | |

*Estimates = Effect size; CI (Confidence Interval) = Range of true effect; σ² (Residual Variance) = Unexplained variability; τ00 (Intercept Variance) = Group variability; ICC (Intraclass Correlation Coefficient) = Group effect proportion; Marginal R² = Fixed effects variance; Conditional R² = Total variance (fixed + random). The models include an interaction term between temperature and month to test for time-varying associations.*

# **Sensitivity Analysis (Robustness check)**

## 5. Models with single day temperatures

**Table 7a**.

*Linear Models of Outdoor Temperature and Sleep Outcomes.*

|  | **TST** | | | **SE** | | | **Sleep Quality** | | |
| --- | --- | --- | --- | --- | --- | --- | --- | --- | --- |
| *Predictors* | *Estimates* | *CI* | *p* | *Estimates* | *CI* | *p* | *Estimates* | *CI* | *p* |
| (Intercept) | 402.95 | 193.98, 611.93 | **<0.001** | 96.86 | 83.51, 110.22 | **<0.001** | 11.40 | 6.67, 16.13 | **<0.001** |
| Lagged Outcome | 0.02 | -0.05, 0.09 | 0.548 | 0.02 | -0.05, 0.09 | 0.569 | 0.21 | 0.13, 0.30 | **<0.001** |
| Workday | 1.85 | -11.95, 15.66 | 0.792 | 0.73 | 0.08, 1.38 | **0.027** | -0.18 | -2.36, 1.99 | 0.868 |
| Age | 2.80 | -1.38, 6.98 | 0.189 | 0.05 | -0.19, 0.29 | 0.663 | -0.03 | -0.10, 0.05 | 0.458 |
| Sex | 6.93 | -42.05, 55.91 | 0.781 | -0.61 | -3.43, 2.22 | 0.673 | -0.84 | -1.72, 0.04 | 0.062 |
| Max Outdoor T | -3.27 | -4.93, -1.61 | **<0.001** | -0.10 | -0.18, -0.03 | **0.009** | -0.11 | -0.16, -0.07 | **<0.001** |
| Steps (thousands) | -1.93 | -3.30, -0.56 | **0.006** | -0.04 | -0.10, 0.03 | 0.259 | -0.03 | -0.07, 0.02 | 0.237 |
| Outdoor Humidity | -0.07 | -0.72, 0.57 | 0.826 | -0.02 | -0.05, 0.01 | 0.206 | -0.00 | -0.02, 0.01 | 0.629 |
| **Additional Information** | | | | | | | | | |
| σ^2^ | 5260.02 | | | 11.24 | | | 2.34 | | |
| τ_00_ | 1192.75 _participant_ | | | 4.08 _participant_ | | | 0.34 _participant_ | | |
| ICC | 0.18 | | | 0.27 | | | 0.13 | | |
| N | 13 _participant_ | | | 13 _participant_ | | | 13 _participant_ | | |
| Observations | 880 | | | 867 | | | 529 | | |
| Marginal R^2^ / Conditional R^2^ | 0.048 / 0.224 | | | 0.023 / 0.283 | | | 0.180 / 0.284 | | |

*Estimates = Effect size; CI (Confidence Interval) = Range of true effect; σ² (Residual Variance) = Unexplained variability; τ00 (Intercept Variance) = Group variability; ICC (Intraclass Correlation Coefficient) = Group effect proportion; Marginal R² = Fixed effects variance; Conditional R² = Total variance (fixed + random).*

**Table 7b.**

*Linear Models of Indoor Temperature and Sleep Outcomes*

|  | **TST** | | | **SE** | | | **Sleep Quality** | | |
| --- | --- | --- | --- | --- | --- | --- | --- | --- | --- |
| *Predictors* | *Estimates* | *CI* | *p* | *Estimates* | *CI* | *p* | *Estimates* | *CI* | *p* |
| (Intercept) | 439.25 | 230.15, 648.35 | **<0.001** | 99.23 | 86.73, 111.73 | **<0.001** | 14.98 | 9.36, 20.59 | **<0.001** |
| Lagged Outcome | 0.02 | -0.05, 0.08 | 0.615 | 0.01 | -0.05, 0.08 | 0.690 | 0.21 | 0.13, 0.29 | **<0.001** |
| Workday | -1.15 | -13.83, 11.54 | 0.859 | 0.62 | 0.04, 1.20 | **0.037** | -0.34 | -2.53, 1.85 | 0.760 |
| Age | 2.43 | -1.51, 6.37 | 0.226 | 0.03 | -0.18, 0.25 | 0.777 | -0.04 | -0.13, 0.05 | 0.391 |
| Sex | 9.12 | -36.74, 54.97 | 0.697 | -0.59 | -3.11, 1.93 | 0.645 | -0.62 | -1.68, 0.44 | 0.253 |
| Max Indoor T | -3.33 | -5.62, -1.03 | **0.005** | -0.16 | -0.26, -0.05 | **0.003** | -0.18 | -0.25, -0.11 | **<0.001** |
| Steps (thousands) | -2.20 | -3.56, -0.84 | **0.002** | -0.03 | -0.09, 0.04 | 0.401 | -0.02 | -0.06, 0.02 | 0.376 |
| Indoor Humidity | -0.62 | -1.70, 0.46 | 0.259 | -0.02 | -0.07, 0.03 | 0.395 | -0.04 | -0.07, -0.01 | **0.005** |
| **Additional Information** | | | | | | | | | |
| σ^2^ | 5611.28 | | | 11.39 | | | 2.36 | | |
| τ_00_ | 1047.83 _participant_ | | | 3.25 _participant_ | | | 0.53 _participant_ | | |
| ICC | 0.16 | | | 0.22 | | | 0.18 | | |
| N | 13 _participant_ | | | 13 _participant_ | | | 13 _participant_ | | |
| Observations | 964 | | | 957 | | | 546 | | |
| Marginal R^2^ / Conditional R^2^ | 0.037 / 0.188 | | | 0.027 / 0.243 | | | 0.163 / 0.317 | | |

*Estimates = Effect size; CI (Confidence Interval) = Range of true effect; σ² (Residual Variance) = Unexplained variability; τ00 (Intercept Variance) = Group variability; ICC (Intraclass Correlation Coefficient) = Group effect proportion; Marginal R² = Fixed effects variance; Conditional R² = Total variance (fixed + random).*

**Table 8a**.

*Linear Models of Outdoor Temperature and Sleep Outcomes by Month.*

|  | **TST** | | | **SE** | | | **Sleep Quality** | | |
| --- | --- | --- | --- | --- | --- | --- | --- | --- | --- |
| *Predictors* | *Estimates* | *CI* | *p* | *Estimates* | *CI* | *p* | *Estimates* | *CI* | *p* |
| (Intercept) | 397.65 | 186.67, 608.63 | **<0.001** | 97.97 | 84.29, 111.65 | **<0.001** | 11.76 | 7.08, 16.43 | **<0.001** |
| Lagged Outcome | -0.00 | -0.07, 0.06 | 0.909 | 0.00 | -0.07, 0.07 | 0.957 | 0.20 | 0.12, 0.28 | **<0.001** |
| Workday | 1.53 | -12.31, 15.36 | 0.829 | 0.79 | 0.13, 1.45 | **0.018** | -0.22 | -2.38, 1.95 | 0.844 |
| Age | 2.47 | -1.75, 6.69 | 0.251 | 0.06 | -0.19, 0.30 | 0.644 | -0.03 | -0.11, 0.04 | 0.361 |
| Sex | 6.74 | -42.71, 56.18 | 0.789 | -0.64 | -3.53, 2.26 | 0.666 | -0.84 | -1.71, 0.02 | 0.056 |
| Month2 [July] | -1.63 | -14.05, 10.78 | 0.796 | -0.60 | -1.19, -0.02 | **0.044** | 0.22 | -0.11, 0.55 | 0.196 |
| Month2 [Aug] | 24.69 | 11.40, 37.98 | **<0.001** | -0.68 | -1.31, -0.05 | **0.033** | 0.62 | 0.25, 0.98 | **0.001** |
| Max Outdoor T | -3.41 | -5.20, -1.63 | **<0.001** | -0.07 | -0.16, 0.01 | 0.103 | -0.13 | -0.18, -0.08 | **<0.001** |
| Steps (thousands) | -1.72 | -3.10, -0.35 | **0.014** | -0.05 | -0.12, 0.02 | 0.138 | -0.02 | -0.06, 0.02 | 0.291 |
| Outdoor Humidity | 0.26 | -0.40, 0.92 | 0.436 | -0.02 | -0.05, 0.01 | 0.201 | 0.00 | -0.02, 0.02 | 0.952 |
| **Additional Information** | | | | | | | | | |
| σ^2^ | 5163.47 | | | 11.28 | | | 2.30 | | |
| τ_00_ | 1217.87 _participant_ | | | 4.30 _participant_ | | | 0.33 _participant_ | | |
| ICC | 0.19 | | | 0.28 | | | 0.12 | | |
| N | 13 _participant_ | | | 13 _participant_ | | | 13 _participant_ | | |
| Observations | 859 | | | 840 | | | 528 | | |
| Marginal R^2^ / Conditional R^2^ | 0.064 / 0.242 | | | 0.028 / 0.296 | | | 0.198 / 0.298 | | |

*Estimates = Effect size; CI (Confidence Interval) = Range of true effect; σ² (Residual Variance) = Unexplained variability; τ00 (Intercept Variance) = Group variability; ICC (Intraclass Correlation Coefficient) = Group effect proportion; Marginal R² = Fixed effects variance; Conditional R² = Total variance (fixed + random).*

**Table 8b**.

*Linear Models of Indoor Temperature and Sleep Outcomes by Month.*

|  | **TST** | | | **SE** | | | **Sleep Quality** | | |
| --- | --- | --- | --- | --- | --- | --- | --- | --- | --- |
| *Predictors* | *Estimates* | *CI* | *p* | *Estimates* | *CI* | *p* | *Estimates* | *CI* | *p* |
| (Intercept) | 404.61 | 193.25, 615.98 | **<0.001** | 100.75 | 87.61, 113.88 | **<0.001** | 14.55 | 8.78, 20.32 | **<0.001** |
| Lagged Outcome | -0.01 | -0.08, 0.06 | 0.782 | 0.00 | -0.06, 0.06 | 0.995 | 0.20 | 0.12, 0.28 | **<0.001** |
| Workday | 0.13 | -12.62, 12.87 | 0.985 | 0.66 | 0.07, 1.25 | **0.029** | -0.38 | -2.56, 1.81 | 0.736 |
| Age | 2.18 | -1.76, 6.12 | 0.278 | 0.04 | -0.19, 0.27 | 0.726 | -0.04 | -0.14, 0.05 | 0.356 |
| Sex | 10.53 | -35.39, 56.44 | 0.653 | -0.65 | -3.30, 2.00 | 0.631 | -0.60 | -1.71, 0.51 | 0.291 |
| Month2 [July] | 1.92 | -12.06, 15.89 | 0.788 | -0.50 | -1.14, 0.14 | 0.123 | 0.29 | -0.08, 0.66 | 0.124 |
| Month2 [Aug] | 25.74 | 9.17, 42.31 | **0.002** | -0.79 | -1.55, -0.03 | **0.040** | 0.57 | 0.12, 1.02 | **0.013** |
| Max Indoor T | -4.28 | -6.94, -1.62 | **0.002** | -0.10 | -0.22, 0.02 | 0.107 | -0.21 | -0.29, -0.13 | **<0.001** |
| Steps (thousands) | -2.24 | -3.60, -0.88 | **0.001** | -0.03 | -0.10, 0.03 | 0.282 | -0.02 | -0.06, 0.02 | 0.421 |
| Indoor Humidity | 0.50 | -0.75, 1.74 | 0.435 | -0.05 | -0.10, 0.01 | 0.104 | -0.02 | -0.05, 0.01 | 0.229 |
| **Additional Information** | | | | | | | | | |
| σ^2^ | 5533.19 | | | 11.47 | | | 2.34 | | |
| τ_00_ | 1048.18 _participant_ | | | 3.61 _participant_ | | | 0.59 _participant_ | | |
| ICC | 0.16 | | | 0.24 | | | 0.20 | | |
| N | 13 _participant_ | | | 13 _participant_ | | | 13 _participant_ | | |
| Observations | 939 | | | 924 | | | 545 | | |
| Marginal R^2^ / Conditional R^2^ | 0.051 / 0.202 | | | 0.027 / 0.260 | | | 0.173 / 0.339 | | |

*Estimates = Effect size; CI (Confidence Interval) = Range of true effect; σ² (Residual Variance) = Unexplained variability; τ00 (Intercept Variance) = Group variability; ICC (Intraclass Correlation Coefficient) = Group effect proportion; Marginal R² = Fixed effects variance; Conditional R² = Total variance (fixed + random).*

**Table 9a**.

*Linear Models of Outdoor Temperature and Sleep Outcomes including interaction between Temperature and Month.*

|  | **TST** | | | **SE** | | | **Sleep Quality** | | |
| --- | --- | --- | --- | --- | --- | --- | --- | --- | --- |
| *Predictors* | *Estimates* | *CI* | *p* | *Estimates* | *CI* | *p* | *Estimates* | *CI* | *p* |
| (Intercept) | 402.41 | 175.86, 628.96 | **0.001** | 99.33 | 85.10, 113.56 | **<0.001** | 8.55 | 3.40, 13.70 | **0.001** |
| Lagged Outcome | -0.01 | -0.07, 0.06 | 0.882 | 0.00 | -0.07, 0.07 | 0.945 | 0.18 | 0.10, 0.26 | **<0.001** |
| Workday | 1.35 | -12.50, 15.20 | 0.848 | 0.80 | 0.14, 1.45 | **0.018** | -0.26 | -2.42, 1.89 | 0.811 |
| Age | 2.49 | -1.73, 6.72 | 0.247 | 0.06 | -0.19, 0.30 | 0.646 | -0.04 | -0.11, 0.04 | 0.336 |
| Sex | 6.69 | -42.78, 56.15 | 0.791 | -0.64 | -3.53, 2.24 | 0.661 | -0.86 | -1.72, 0.00 | 0.051 |
| Month2 [July] | 52.23 | -70.11, 174.57 | 0.402 | -5.16 | -10.92, 0.59 | 0.079 | 2.79 | -0.46, 6.05 | 0.092 |
| Month2 [Aug] | -0.73 | -106.20, 104.74 | 0.989 | -1.35 | -6.30, 3.61 | 0.594 | 5.50 | 2.40, 8.60 | **0.001** |
| Max Outdoor T | -3.46 | -6.56, -0.35 | **0.029** | -0.09 | -0.20, 0.01 | 0.085 | -0.03 | -0.11, 0.06 | 0.579 |
| Steps (thousands) | -1.84 | -3.22, -0.45 | **0.010** | -0.05 | -0.11, 0.02 | 0.189 | -0.03 | -0.07, 0.02 | 0.232 |
| Outdoor Humidity | 0.22 | -0.45, 0.89 | 0.513 | -0.02 | -0.05, 0.01 | 0.193 | 0.01 | -0.01, 0.02 | 0.511 |
| Month2 [July] * max outdoor T | -1.71 | -5.75, 2.33 | 0.406 | 0.12 | -0.05, 0.30 | 0.158 | -0.09 | -0.20, 0.02 | 0.100 |
| Month2 [Aug] * max outdoor T | 0.79 | -2.71, 4.29 | 0.658 | -0.03 | -0.19, 0.14 | 0.757 | -0.16 | -0.26, -0.06 | **0.002** |
| **Additional Information** | | | | | | | | | |
| σ^2^ | 5164.52 | | | 11.27 | | | 2.27 | | |
| τ_00_ | 1218.72 _participant_ | | | 4.27 _participant_ | | | 0.33 _participant_ | | |
| ICC | 0.19 | | | 0.27 | | | 0.13 | | |
| N | 13 _participant_ | | | 13 _participant_ | | | 13 _participant_ | | |
| Observations | 859 | | | 840 | | | 528 | | |
| Marginal R^2^ / Conditional R^2^ | 0.066 / 0.244 | | | 0.030 / 0.296 | | | 0.210 / 0.309 | | |

*Estimates = Effect size; CI (Confidence Interval) = Range of true effect; σ² (Residual Variance) = Unexplained variability; τ00 (Intercept Variance) = Group variability; ICC (Intraclass Correlation Coefficient) = Group effect proportion; Marginal R² = Fixed effects variance; Conditional R² = Total variance (fixed + random). The models include an interaction term between temperature and month to test for time-varying associations.*

**Table 9b**.

*Linear Models of Indoor Temperature and Sleep Outcomes including interaction between Temperature and Month.*

|  | **TST** | | | **SE** | | | **Sleep Quality** | | |
| --- | --- | --- | --- | --- | --- | --- | --- | --- | --- |
| *Predictors* | *Estimates* | *CI* | *p* | *Estimates* | *CI* | *p* | *Estimates* | *CI* | *p* |
| (Intercept) | 428.03 | 204.21, 651.85 | **<0.001** | 101.74 | 88.04, 115.45 | **<0.001** | 11.50 | 5.58, 17.43 | **<0.001** |
| Lagged Outcome | -0.01 | -0.08, 0.06 | 0.750 | -0.00 | -0.06, 0.06 | 0.982 | 0.17 | 0.09, 0.25 | **<0.001** |
| Workday | 0.54 | -12.21, 13.28 | 0.934 | 0.65 | 0.06, 1.24 | **0.032** | -0.30 | -2.47, 1.87 | 0.786 |
| Age | 2.15 | -1.73, 6.04 | 0.277 | 0.04 | -0.19, 0.27 | 0.729 | -0.04 | -0.14, 0.05 | 0.358 |
| Sex | 11.05 | -34.21, 56.32 | 0.632 | -0.66 | -3.33, 2.00 | 0.625 | -0.63 | -1.71, 0.46 | 0.258 |
| Month2 [July] | 78.42 | -67.19, 224.04 | 0.291 | -4.01 | -10.68, 2.66 | 0.239 | 4.21 | 0.38, 8.05 | **0.031** |
| Month2 [Aug] | -27.32 | -159.66, 105.03 | 0.686 | -1.84 | -7.92, 4.24 | 0.553 | 6.92 | 3.15, 10.68 | **<0.001** |
| Max Indoor T | -4.64 | -8.23, -1.04 | **0.011** | -0.14 | -0.30, 0.03 | 0.103 | -0.10 | -0.20, -0.00 | **0.042** |
| Steps (thousands) | -2.36 | -3.73, -0.99 | **0.001** | -0.03 | -0.10, 0.03 | 0.325 | -0.02 | -0.06, 0.02 | 0.304 |
| Indoor Humidity | 0.29 | -1.00, 1.58 | 0.656 | -0.05 | -0.11, 0.01 | 0.118 | -0.01 | -0.05, 0.02 | 0.457 |
| Month2 [July] * Max Indoor T | -2.80 | -8.26, 2.67 | 0.316 | 0.13 | -0.12, 0.38 | 0.300 | -0.15 | -0.30, -0.01 | **0.037** |
| Month2 [Aug] * Max Indoor T | 1.90 | -3.02, 6.83 | 0.448 | 0.04 | -0.18, 0.27 | 0.715 | -0.24 | -0.38, -0.10 | **0.001** |
| **Additional Information** | | | | | | | | | |
| σ^2^ | 5532.34 | | | 11.48 | | | 2.30 | | |
| τ_00_ | 1016.07 _participant_ | | | 3.64 _participant_ | | | 0.56 _participant_ | | |
| ICC | 0.16 | | | 0.24 | | | 0.20 | | |
| N | 13 _participant_ | | | 13 _participant_ | | | 13 _participant_ | | |
| Observations | 939 | | | 924 | | | 545 | | |
| Marginal R^2^ / Conditional R^2^ | 0.055 / 0.202 | | | 0.027 / 0.262 | | | 0.189 / 0.348 | | |

*Estimates = Effect size; CI (Confidence Interval) = Range of true effect; σ² (Residual Variance) = Unexplained variability; τ00 (Intercept Variance) = Group variability; ICC (Intraclass Correlation Coefficient) = Group effect proportion; Marginal R² = Fixed effects variance; Conditional R² = Total variance (fixed + random). The models include an interaction term between temperature and month to test for time-varying associations.*

## 6. Models adjusted for daylight duration

**Table 10a**.

*Linear Models of Outdoor Temperature and Sleep Outcomes,* adjusted for daylight duration.

|  | **TST** | | | **SE** | | | **Sleep Quality** | | |
| --- | --- | --- | --- | --- | --- | --- | --- | --- | --- |
| *Predictors* | *Est.* | *CI* | *p* | *Est.* | *CI* | *p* | *Est.* | *CI* | *p* |
| (Intercept) | 382.82 | 173.37, 592.28 | **<0.001** | 96.09 | 82.66, 109.53 | <0.001 | 10.77 | 6.13, 15.42 | **<0.001** |
| Lagged Outcome | −0.01 | −0.07, 0.06 | 0.878 | 0.02 | −0.05, 0.08 | 0.625 | 0.2 | 0.12, 0.29 | **<0.001** |
| Workday [1] | 1.56 | −12.14, 15.26 | 0.823 | 0.73 | 0.08, 1.38 | **0.027** | −0.37 | −2.54, 1.80 | 0.741 |
| Daylight (hours) | −20.45 | −28.69, −12.21 | **<0.001** | 0.13 | −0.25, 0.52 | 0.5 | −0.41 | −0.65, −0.17 | **0.001** |
| Age | 2.46 | −1.76, 6.67 | 0.253 | 0.06 | −0.19, 0.30 | 0.655 | −0.04 | −0.11, 0.04 | 0.336 |
| Sex | 6.38 | −42.93, 55.68 | 0.8 | −0.60 | −3.44, 2.25 | 0.681 | −0.85 | −1.70, 0.01 | 0.052 |
| Max Outdoor T 3day MA | −3.07 | −4.80, −1.34 | **0.001** | −0.08 | −0.17, −0.00 | **0.043** | −0.11 | −0.16, −0.06 | **<0.001** |
| Steps (thousands) | −1.79 | −3.15, −0.42 | **0.01** | −0.04 | −0.11, 0.02 | 0.216 | −0.02 | −0.06, 0.02 | 0.281 |
| Indoor Humidity | 0.47 | −0.14, 1.08 | 0.13 | −0.01 | −0.04, 0.02 | 0.353 | 0.01 | −0.01, 0.03 | 0.251 |
| **Additional Information** | | | | | | | | | |
| σ^2^ | 5177.09 | | | 11.27 | | | 2.33 | | |
| τ_00_ | 1211.45 _participant_ | | | 4.14 _participant_ | | | 0.32 _participant_ | | |
| ICC | 0.19 | | | 0.27 | | | 0.12 | | |
| N | 13 _participant_ | | | 13 _participant_ | | | 13 _participant_ | | |
| Observations | 880 | | | 867 | | | 529 | | |
| Marginal R^2^ / Conditional R^2^ | 0.061/0.239 | | | 0.022/0.285 | | | 0.192/0.289 | | |

*Estimates = Effect size; CI (Confidence Interval) = Range of true effect; σ² (Residual Variance) = Unexplained variability; τ00 (Intercept Variance) = Group variability; ICC (Intraclass Correlation Coefficient) = Group effect proportion; Marginal R² = Fixed effects variance; Conditional R² = Total variance (fixed + random).*

**Table 10b.**

*Linear Models of Indoor Temperature and Sleep Outcomes,* adjusted for daylight duration.

|  |  | | **TST** | | | **SE** | | | **Sleep Quality** | | |
| --- | --- | --- | --- | --- | --- | --- | --- | --- | --- | --- | --- |
| *Predictors* |  | | *Est.* | *CI* | *p* | *Est.* | *CI* | *p* | *Est.* | *CI* | *p* |
| (Intercept) |  | | 403.01 | 187.34, 618.69 | <0.001 | 101.53 | 88.71, 114.34 | <0.001 | 14.46 | 8.65, 20.26 | <0.001 |
| Lagged Outcome |  | | -0.01 | -0.07, 0.06 | 0.848 | 0.01 | -0.05, 0.07 | 0.21 | 0.13, 0.29 | <0.001 | 0.21 |
| Workday [1] |  | | -1.13 | -13.70, 11.44 | 0.86 | 0.59 | 0.01, 1.18 | 0.045 | -0.53 | -2.73, 1.66 | 0.635 |
| Daylight (hours) |  | | -22.55 | -31.92, -13.19 | <0.001 | 0.21 | -0.22, 0.63 | 0.342 | -0.26 | -0.54, 0.01 | 0.055 |
| Age |  | | 2.21 | -1.74, 6.17 | 0.272 | 0.03 | -0.18, 0.25 | 0.761 | -0.04 | -0.13, 0.05 | 0.356 |
| Sex |  | | 9.67 | -36.39, 55.73 | 0.68 | -0.57 | -3.09, 1.96 | 0.661 | -0.65 | -1.71, 0.40 | 0.226 |
| Max Indoor T 3day MA |  | | -4.2 | -6.70, -1.71 | 0.001 | -0.18 | -0.29, -0.06 | 0.002 | -0.18 | -0.25, -0.10 | <0.001 |
| Steps (thousands) |  | | -2.23 | -3.58, -0.89 | 0.001 | -0.03 | -0.09, 0.03 | 0.355 | -0.02 | -0.06, 0.02 | 0.392 |
| Indoor Humidity |  | | 0.65 | -0.62, 1.91 | 0.319 | -0.05 | -0.11, 0.01 | 0.11 | -0.03 | -0.06, 0.01 | 0.126 |
| **Additional Information** | |  | | | | | | | | | |
| σ^2^ |  | | 5502.49 | | | 11.38 | | | 2.37 | | |
| τ_00_ |  | | 1058.50 _participant_ | | | 3.27 _participant_ | | | 0.52 _participant_ | | |
| ICC |  | | 0.16 | | | 0.22 | | | 0.18 | | |
| N |  | | 13 _participant_ | | | 13 _participant_ | | | 13 _participant_ | | |
| Observations |  | | 963 | | | 956 | | | 546 | | |
| Marginal R^2^ / Conditional R^2^ |  | | 0.054 / 0.207 | | | 0.030 / 0.246 | | | 0.164 / 0.315 | | |

*Estimates = Effect size; CI (Confidence Interval) = Range of true effect; σ² (Residual Variance) = Unexplained variability; τ00 (Intercept Variance) = Group variability; ICC (Intraclass Correlation Coefficient) = Group effect proportion; Marginal R² = Fixed effects variance; Conditional R² = Total variance (fixed + random).*

## 7. Models with both temperatures combined

**Table 11a.**

*Linear Models of Outdoor and Indoor Temperature and Sleep Outcomes*.

|  |  | | **TST** | | | **SE** | | | **Sleep Quality** | | |
| --- | --- | --- | --- | --- | --- | --- | --- | --- | --- | --- | --- |
| *Predictors* |  | | *Est.* | *CI* | *p* | *Est.* | *CI* | *p* | *Est.* | *CI* | *p* |
| (Intercept) |  | | 407.7 | 177.30, 638.10 | **0.001** | 100.9 | 87.36, 114.43 | **<0.001** | 14.67 | 9.44, 19.89 | **<0.001** |
| Lagged Outcome |  | | 0.02 | -0.05, 0.09 | 0.592 | 0.01 | -0.05, 0.08 | 0.701 | 0.21 | 0.12, 0.29 | **<0.001** |
| Workday [1] |  | | 1.25 | -12.64, 15.13 | 0.86 | 0.74 | 0.09, 1.38 | **0.026** | -0.15 | -2.30, 2.01 | 0.893 |
| Age |  | | 2.81 | -1.46, 7.09 | 0.197 | 0.04 | -0.19, 0.26 | 0.742 | -0.03 | -0.10, 0.04 | 0.384 |
| Sex |  | | 7.29 | -42.90, 57.48 | 0.776 | -0.35 | -3.01, 2.32 | 0.799 | -0.71 | -1.57, 0.15 | 0.107 |
| Max Outdoor T 3day MA |  | | -3.81 | -7.06, -0.56 | **0.021** | 0.08 | -0.07, 0.23 | 0.284 | -0.11 | -0.19, -0.02 | **0.015** |
| Max Indoor T 3day MA |  | | 1.78 | -2.83, 6.39 | 0.448 | -0.3 | -0.51, -0.08 | **0.007** | -0.03 | -0.16, 0.10 | 0.667 |
| Steps (thousands) |  | | -1.81 | -3.22, -0.40 | **0.012** | -0.05 | -0.11, 0.02 | 0.161 | -0.02 | -0.06, 0.02 | 0.272 |
| Indoor Humidity |  | | 0.59 | -0.13, 1.31 | 0.109 | 0 | -0.03, 0.04 | 0.859 | 0.03 | 0.01, 0.05 | **0.009** |
| **Additional Information** | |  | | | | | | | | | |
| σ^2^ |  | | 5300.07 | | | 11.2 | | | 2.29 | | |
| τ_00_ |  | | 1246.24 _participant_ | | | 3.58 _participant_ | | | 0.32 _participant_ | | |
| ICC |  | | 0.19 | | | 0.24 | | | 0.12 | | |
| N |  | | 13 _participant_ | | | 13 _participant_ | | | 13 _participant_ | | |
| Observations |  | | 873 | | | 867 | | | 529 | | |
| Marginal R^2^ / Conditional R^2^ |  | | 0.049 / 0.230 | | | 0.037 / 0.270 | | | 0.208 / 0.304 | | |

*Estimates = Effect size; CI (Confidence Interval) = Range of true effect; σ² (Residual Variance) = Unexplained variability; τ00 (Intercept Variance) = Group variability; ICC (Intraclass Correlation Coefficient) = Group effect proportion; Marginal R² = Fixed effects variance; Conditional R² = Total variance (fixed + random).*

**Table 11b.**

Collinearity diagnostics for indoor and outdoor temperature terms

| **outcome** | **Term** | **VIF** | **VIF 95% CI** | **Tolerance** | **Tolerance 95% CI** |
| --- | --- | --- | --- | --- | --- |
| TST | max_outdoor_T_3day_MA | 5.03 | 4.480, 5.668 | 0.199 | 0.176, 0.223 |
| TST | max_indoor_T_3day_MA | 5.188 | 4.618, 5.848 | 0.193 | 0.171, 0.217 |
| SE | max_outdoor_T_3day_MA | 5.131 | 4.566, 5.785 | 0.195 | 0.173, 0.219 |
| SE | max_indoor_T_3day_MA | 5.272 | 4.690, 5.946 | 0.19 | 0.168, 0.213 |
| Sleep Quality | max_outdoor_T_3day_MA | 4.945 | 4.272, 5.757 | 0.202 | 0.174, 0.234 |
| Sleep Quality | max_indoor_T_3day_MA | 4.811 | 4.158, 5.599 | 0.208 | 0.179, 0.240 |

*VIF = variance inflation factor; CI = confidence interval. Tolerance was calculated as 1/VIF. Diagnostics were obtained from models including indoor and outdoor 3-day moving-average maximum temperature simultaneously.*

## 8. Models accounting for individual heterogeneity

### Random slope models

**Table 12a**.

*Linear Models of Outdoor Temperature and Sleep Outcomes with random slopes*.

|  | **TST** | | | **SE** | | | **Sleep Quality** | | |
| --- | --- | --- | --- | --- | --- | --- | --- | --- | --- |
| *Predictors* | *Est.* | *CI* | *p* | *Est.* | *CI* | *p* | *Est.* | *CI* | *p* |
| (Intercept) | 358.21 | 148.21, 568.22 | **0.001** | 96.52 | 82.93, 110.11 | **<0.001** | 11.97 | 6.46, 17.49 | **<0.001** |
| Lagged Outcome | 0.02 | -0.05, 0.09 | 0.577 | 0.01 | -0.06, 0.08 | 0.81 | 0.17 | 0.09, 0.25 | **<0.001** |
| Workday [1] | 1.76 | -12.12, 15.64 | 0.803 | 0.73 | 0.09, 1.38 | **0.026** | -0.46 | -2.61, 1.69 | 0.672 |
| Age | 2.6 | -1.63, 6.82 | 0.228 | 0.07 | -0.18, 0.31 | 0.598 | -0.03 | -0.11, 0.05 | 0.443 |
| Sex | 4.11 | -45.04, 53.27 | 0.87 | -0.66 | -3.50, 2.19 | 0.65 | -0.94 | -1.87, -0.01 | **0.047** |
| Max Outdoor T 3day MA | -2.18 | -3.90, -0.46 | **0.013** | -0.08 | -0.18, 0.02 | 0.129 | -0.12 | -0.21, -0.02 | **0.015** |
| Steps (thousands) | -1.98 | -3.36, -0.60 | **0.005** | -0.04 | -0.11, 0.02 | 0.223 | -0.03 | -0.07, 0.01 | 0.197 |
| Outdoor Humidity | 0.28 | -0.33, 0.89 | 0.361 | -0.01 | -0.04, 0.01 | 0.336 | 0 | -0.01, 0.02 | 0.733 |
| **Additional Information** | | | | | | | | | |
| σ^2^ | 5311.1 | | | 11.13 | | | 2.26 | | |
| τ_00_ | 614.39 _participant_ | | | 11.90 _participant_ | | | 22.08 _participant_ | | |
| τ11 | 0.11 _participant.max_outdoor_T_3day_MA_ | | | 0.01 _participant.max_outdoor_T_3day_MA_ | | | 0.02 _participant_._max_outdoor_T_3day_MA_ | | |
| ρ01 | 1.00 _participant_ | | | -0.82 _participant_ | | | -0.99 _participant_ | | |
| ICC | 0.19 | | | 0.28 | | | 0.23 | | |
| N | 13 _participant_ | | | 13 _participant_ | | | 13 _participant_ | | |
| Observations | 880 | | | 867 | | | 529 | | |
| Marginal R^2^ / Conditional R^2^ | 0.040 / 0.220 | | | 0.023 / 0.299 | | | 0.169 / 0.360 | | |

*Estimates = Effect size; CI (Confidence Interval) = Range of true effect; σ² (Residual Variance) = Unexplained variability; τ00 (Random Intercept Variance) = Between-participant variability in baseline sleep levels; τ11 (Random Slope Variance) = Between-participant variability in the temperature–sleep association; ρ01 (Intercept–Slope Correlation) = Correlation between participant-specific baseline sleep levels and temperature slopes;ICC (Intraclass Correlation Coefficient) = Group effect proportion; Marginal R² = Fixed effects variance; Conditional R² = Total variance (fixed + random).*

**Table 12b.**

*Linear Models of Indoor Temperature and Sleep Outcomes with random slopes*.

|  |  | **TST** | | | **SE** | | | **Sleep Quality** | | |
| --- | --- | --- | --- | --- | --- | --- | --- | --- | --- | --- |
| *Predictors* |  | *Est.* | *CI* | *p* | *Est.* | *CI* | *p* | *Est.* | *CI* | *p* |
| (Intercept) |  | 473.61 | 244.76, 702.46 | **<0.001** | 103.58 | 90.97, 116.20 | **<0.001** | 17.74 | 11.27, 24.22 | **<0.001** |
| Lagged Outcome |  | 0.01 | -0.06, 0.07 | 0.796 | 0 | -0.06, 0.06 | 0.947 | 0.17 | 0.08, 0.25 | **<0.001** |
| Workday [1] |  | -0.83 | -13.50, 11.84 | 0.898 | 0.64 | 0.06, 1.22 | **0.031** | -0.46 | -2.62, 1.70 | 0.678 |
| Age |  | 2.18 | -1.88, 6.23 | 0.292 | 0 | -0.21, 0.21 | 0.983 | -0.06 | -0.15, 0.03 | 0.169 |
| Sex |  | 11.73 | -35.64, 59.10 | 0.627 | -0.78 | -3.04, 1.47 | 0.496 | -0.7 | -1.76, 0.36 | 0.197 |
| Max Indoor T 3day MA |  | -3.91 | -7.35, -0.48 | **0.025** | -0.2 | -0.33, -0.06 | **0.004** | -0.21 | -0.33, -0.08 | **0.001** |
| Steps (thousands) |  | -2.2 | -3.56, -0.83 | **0.002** | -0.03 | -0.09, 0.03 | 0.363 | -0.02 | -0.07, 0.02 | 0.251 |
| Indoor Humidity |  | -0.82 | -1.98, 0.33 | 0.16 | -0.04 | -0.09, 0.02 | 0.165 | -0.05 | -0.08, -0.02 | **0.001** |
| **Additional Information** | | | | | | | | | | |
| σ^2^ |  | 5561.9 | | | 11.27 | | | 2.29 | | |
| τ_00_ |  | 11922.39 participant | | | 5.60 participant | | | 23.10 participant | | |
| τ11 |  | 14.62 participant.max_indoor_T_3day_MA | | | 0.02 participant.max_indoor_T_3day_MA | | | 0.03 participant.max_indoor_T_3day_MA | | |
| ρ01 |  | -0.95 participant | | | -0.88 participant | | | -0.99 participant | | |
| ICC |  | 0.18 | | | 0.24 | | | 0.25 | | |
| N |  | 13 participant | | | 13 participant | | | 13 participant | | |
| Observations |  | 963 | | | 956 | | | 546 | | |
| Marginal R^2^ / Conditional R^2^ |  | 0.035 / 0.209 | | | 0.032 / 0.262 | | | 0.163 / 0.376 | | |

*Estimates = Effect size; CI (Confidence Interval) = Range of true effect; σ² (Residual Variance) = Unexplained variability; τ00 (Random Intercept Variance) = Between-participant variability in baseline sleep levels; τ11 (Random Slope Variance) = Between-participant variability in the temperature–sleep association; ρ01 (Intercept–Slope Correlation) = Correlation between participant-specific baseline sleep levels and temperature slopes; ICC (Intraclass Correlation Coefficient) = Group effect proportion; Marginal R² = Fixed effects variance; Conditional R² = Total variance (fixed + random).*

**Table 13c.**

Population-average and participant-specific temperature–sleep slopes from random-slope models

| **Outcome** | **Temperature** | **N negative slopes** | **N positive slopes** | **Mean individual slope** | **SD individual slope** | **Min individual slope** | **Max individual slope** | **Fixed-effect slope** | **Model Random slope SD** |
| --- | --- | --- | --- | --- | --- | --- | --- | --- | --- |
| TST | Indoor | 12 | 1 | -3.915 | 2.545 | -7.82 | 0.018 | -3.915 | 3.824 |
| TST | Outdoor | 13 | 0 | -2.181 | 0.29 | -2.564 | -1.465 | -2.181 | 0.332 |
| SE | Indoor | 13 | 0 | -0.197 | 0.103 | -0.429 | -0.108 | -0.197 | 0.134 |
| SE | Outdoor | 12 | 1 | -0.079 | 0.071 | -0.254 | 0.015 | -0.079 | 0.11 |
| Sleep Quality | Indoor | 12 | 1 | -0.209 | 0.131 | -0.411 | 0.003 | -0.209 | 0.166 |
| Sleep Quality | Outdoor | 12 | 1 | -0.119 | 0.091 | -0.246 | 0.019 | -0.119 | 0.144 |

Participants: 13; Fixed-effect slope = Population-average temperature–sleep association; Model-estimated random-slope SD = Estimated between-participant variability in the latent temperature slopes; Mean conditional slope = Average of the 13 participant-specific conditional slopes; SD of conditional slopes = Empirical variability across the 13 extracted conditional slopes; Minimum and maximum conditional slopes = Lowest and highest participant-specific conditional slopes.

### Leave-One-Out analysis (LOO)

**Table 14.**

*Leave-one-participant-out sensitivity analysis across sleep outcomes*

| **Outcome** | **Temperature** | **Full sample estimate** | **Mean LOO estimate** | **Min-max LOO estimate** | **Max absolute change** | **N same direction** | **N CI excluding zero same direction** |
| --- | --- | --- | --- | --- | --- | --- | --- |
| TST | Indoor | -4.20 | -4.20 | -5.06, -3.57 | 0.86 | 13/13 | 1 |
| TST | Outdoor | -3.07 | -3.06 | -3.36, -2.69 | 0.38 | 13/13 | 1 |
| SE | Indoor | -0.18 | -0.18 | -0.21, -0.10 | 0.08 | 13/13 | 1 |
| SE | Outdoor | -0.08 | -0.08 | -0.10, -0.05 | 0.04 | 13/13 | 0 |
| Sleep Quality | Indoor | -0.18 | -0.18 | -0.22, -0.13 | 0.05 | 13/13 | 1 |
| Sleep Quality | Outdoor | -0.11 | -0.11 | -0.13, -0.09 | 0.02 | 13/13 | 1 |

Participants: 13. LOO = leave-one-participant-out models, fitted after excluding each participant in turn. Min–max = range across LOO estimates; max absolute change = largest difference from the full-sample estimate. N same direction = LOO estimates matching the full-sample direction; N CI excluding zero = LOO estimates with a 95% CI excluding zero in that direction.

**Figure 3.**

*Leave-One-Out Analysis*

Points and error bars show the temperature–sleep estimate and 95% CI from each leave-one-participant-out model. Solid horizontal lines indicate the corresponding full-sample estimates; dashed lines indicate the null value.
